# Loss of Skeletal Muscle Mass Is Associated With Reduced Cytotoxic T Cell Abundance and Poor Survival in Advanced Lung Cancer

**DOI:** 10.1002/jcsm.70063

**Published:** 2025-09-10

**Authors:** Xin Nie, Yan Sun, David P. J. van Dijk, Min Deng, Ralph Brecheisen, Zhaoqi Wang, Qingxin Xia, Steven M. W. Olde Damink, Sander S. Rensen

**Affiliations:** ^1^ Department of Radiation Oncology The Affiliated Cancer Hospital of Zhengzhou University & Henan Cancer Hospital Zhengzhou China; ^2^ Department of Surgery, NUTRIM School of Nutrition and Translational Research in Metabolism Maastricht University Maastricht the Netherlands; ^3^ Department of Molecular Cell Biology, Institute for Cancer Research Oslo University Hospital Oslo Norway; ^4^ Department of Radiology The Affiliated Cancer Hospital of Zhengzhou University & Henan Cancer Hospital Zhengzhou China; ^5^ Department of Pathology The Affiliated Cancer Hospital of Zhengzhou University & Henan Cancer Hospital Zhengzhou China; ^6^ Department of General, Visceral, Vascular and Transplantation Surgery University Hospital Essen, Duisberg‐Essen University Essen Germany

**Keywords:** cytotoxic T cell, lung cancer, skeletal muscle mass, survival, TILs

## Abstract

**Background:**

Body composition alterations such as skeletal muscle (SM) loss in cancer patients are associated with poor survival. In turn, immune cell‐driven pathways have been linked to muscle wasting. We aimed to investigate the relationship between body composition, tumour‐infiltrating lymphocytes and survival in patients with advanced lung cancer.

**Methods:**

We studied 200 patients with advanced lung cancer receiving immunotherapy (*n* = 81) or non‐immunotherapy regimens (*n* = 119). Body composition including SM index (SMI) at baseline and longitudinal changes were assessed using computed tomography (CT) scans at the third lumbar vertebra. Associations between body composition parameters and overall survival (OS) were evaluated using Cox regression analysis. The median value of SMI, stratified by sex, was used as the cut‐off to define groups with high and low baseline SMI. Stable SMI was defined by any increase or < 2% decrease per 100 days; loss of SMI was defined by ≥ 2% decrease per 100 days. Logistic regression analysis was applied to investigate the association between SMI and peripheral circulating immune cells. Tumour‐infiltrating lymphocytes were identified by immunohistochemistry, and their relationship with SMI was evaluated.

**Results:**

SMI loss was associated with shorter OS (whole cohort: HR = 2.314, 95% CI = 1.388–3.858, *p* = 0.001; immunotherapy cohort: HR = 3.028, 95% CI = 1.113–8.236, *p* = 0.03; non‐immunotherapy cohort: HR = 2.298, 95% CI = 1.191–4.435, *p* = 0.013). Low baseline SMI was associated with higher CD3^+^ T cell abundance (OR = 1.240, 95% CI = 1.080–1.424, *p* = 0.002) but lower CD3^+^ CD8^+^ T cell abundance (OR = 0.862, 95% CI = 0.762–0.974, *p* = 0.018) in peripheral blood. Subsequent SMI loss during treatment was also significantly associated with higher CD3^+^ T cell counts (OR = 3.414, 95% CI = 1.301–8.961, *p* = 0.013) and lower CD3^+^ CD8^+^ T cell abundance (OR = 0.666, 95% CI = 0.459–0.968, *p* = 0.033). Patients with stable SMI had a higher number of CD8^+^ tumour‐infiltrating lymphocytes than patients with SMI loss (15.4% vs. 7.9%, *p* = 0.036).

**Conclusion:**

SM loss is an independent predictor for survival in patients with advanced lung cancer and is associated with reduced peripheral and tumour‐infiltrating cytotoxic T cell abundance. An inadequate antitumour immune response may contribute to metabolic tissue wasting in cancer.

## 1 Introduction

Lung cancer is one of the leading causes of cancer‐related deaths worldwide, ranking first in mortality rates for men and second for women [1]. In recent years, novel systemic therapies such as immune checkpoint inhibitors (ICIs), which inhibit tumour growth or kill cells by stimulating antitumor immune responses, have been optimized [2, 3]. ICIs are currently widely used to treat patients with solid tumours, especially lung cancer, and have achieved revolutionary treatment effects and long‐term survival [4]. However, a significant proportion of patients do not benefit from ICI treatment, likely because of developing immune tolerance and exhaustion [5, 6]. Although the assessment of tumour cell expression of PD‐L1 and mismatch repair genes (such as MLH1, MSH2, MSH6 and PMS2) as well as microsatellite instability has helped to identify individuals who may clinically benefit from ICI, accurate prediction of immunotherapy outcomes is still challenging [7, 8, 9], and identification of additional prognostic factors is required.

Cancer cachexia–associated body composition alterations are known to complicate the treatment of advanced lung cancer, resulting in a poor treatment response [10]. For example, several studies have shown that sarcopenia, that is, low skeletal muscle (SM) mass, is associated with poor survival of patients receiving chemotherapy [11, 12, 13] or ICIs [14, 15, 16, 17]. Furthermore, loss of SM during treatment is known to be associated with a poor outcome [18, 19].

Interestingly, the tumour immune microenvironment as well as systemic immune factors have been shown to be associated with body composition and immunotherapy responses in cancer patients [20, 21, 22, 23]. Circulating and SM‐infiltrating lymphocytes have recently been reported to be associated with muscle atrophy and cancer‐associated cachexia [24, 25]. However, the link between tumour‐infiltrating lymphocytes (TILs), one of the major response predictors of ICIs [21], and changes in SM mass remains elusive. Therefore, we investigated baseline SM mass and SM alterations during treatment in patients with advanced lung cancer receiving first‐line immunotherapy or nonimmunotherapy regimens. Overall survival (OS) was defined as the primary end‐point. Analyses of TILs and peripheral‐blood lymphocyte subsets were performed in predefined subcohorts and treated as exploratory secondary end‐points intended to explore the interaction between body composition, the immune microenvironment and the response towards ICIs.

## 2 Methods

### 2.1 Patients and Study Design

**FIGURE 1:**
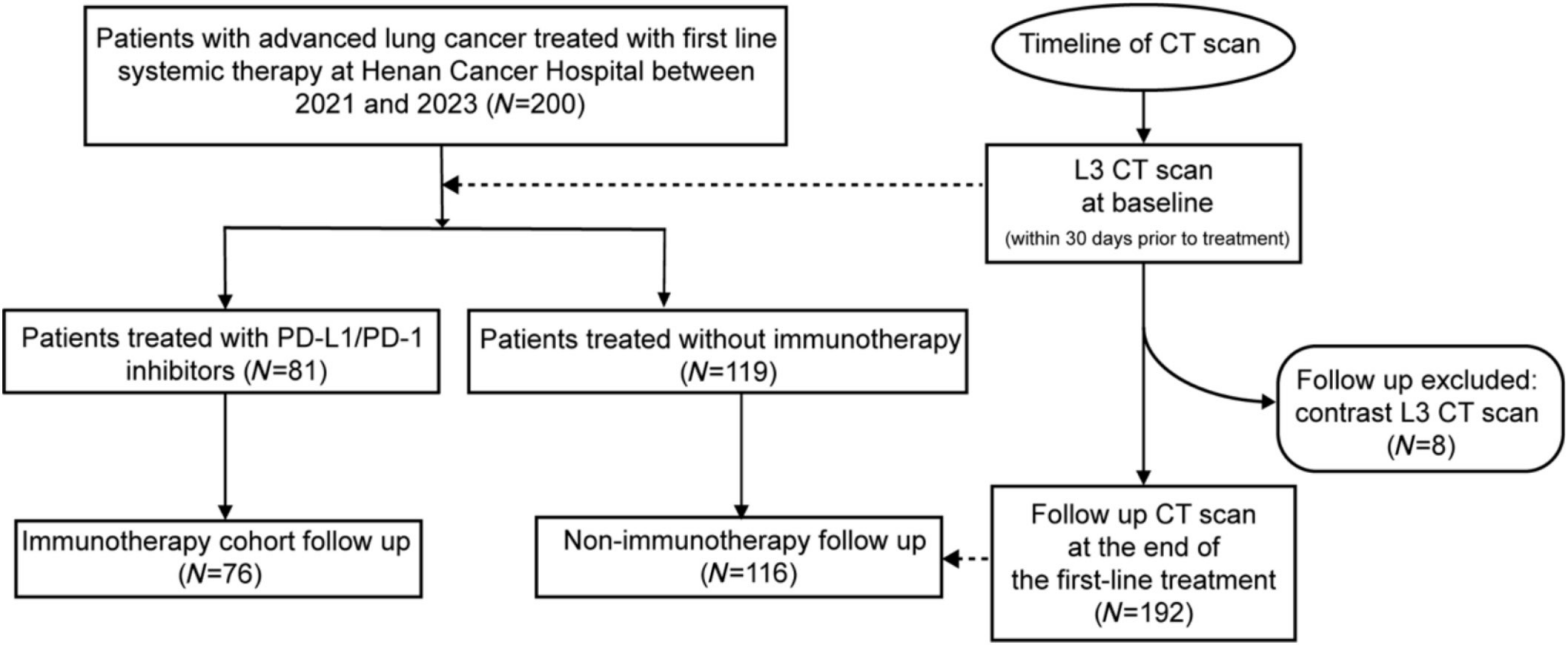
Flowchart showing inclusion and exclusion criteria.

A total of 200 patients with advanced lung cancer presenting at Henan Cancer Hospital between 2021 and 2023 were included. In this retrospective study, 119 patients received first‐line treatment without immunotherapy, and 81 patients received first‐line immunotherapy. Longitudinal radiology data collection and extended follow‐up to monitor disease response to therapy were performed for both cohorts. We collected pretreatment and posttreatment computed tomography (CT) scans to analyse how body composition measures evolved over time (Figure 1) [26]. Subjects were eligible to enrol if the following criteria were met: (1) World Health Organization (WHO) performance score of 0–2; (2) planned first‐line chemotherapy regimen or PD‐L1 treatment; (3) a routine abdominal CT scan was performed within 30 days before the start of treatment (T0), and a follow‐up CT scan (T1) was made at the end of treatment; (4) CT scans were of sufficient quality to perform accurate measurements of tissue area and radiation attenuation (RA); (5) CT scans without contrast agent were available; and (6) clinical characteristics such as age, sex, BMI, height, weight, PD‐L1 expression levels and survival status were recorded. Patients with severe undernutrition (BMI < 16) were excluded to avoid the potential confounding effect of established refractory cachexia.

### 2.2 Body Composition Analysis Based on CT Scan

To assess body composition, CT scans of patients were analysed at the level of the third lumbar vertebra (L3). All L3 images were extracted under the supervision of two radiologists. Tissue cross‐sectional areas and RA were assessed with the validated Mosamatic automatic segmentation tool [27], which uses standard Hounsfield Units (HU) with thresholds of −29 to 150 HU for SM, −150 to −50 HU for visceral adipose tissue (VAT) and −190 to −30 HU for subcutaneous adipose tissue (SAT). The cross‐sectional areas of SM, VAT and SAT were normalized to the patient's height to calculate indexes (cm^2^/m^2^) for SM (SMI), VAT (VATI) and SAT (SATI). Mean RA in HU was assessed for SM (SM‐RA), SAT (SAT‐RA) and VAT (VAT‐RA). To define baseline ‘low’ or ‘high’ SMI, we used sex‐specific median values as cut‐off points. This approach avoids applying non‐representative sarcopenia thresholds derived from Western populations to our Asian cohort, for whom validated cut‐offs are lacking. It also accounts for sex‐related differences in muscle distribution and ensures balanced group sizes to facilitate statistical analysis. Changes in surface area between pretreatment and posttreatment CT scans were expressed as a percentage. This percentage change was divided by the number of days between scans and multiplied by 100 days to obtain a standard measure for all patients (% change per 100 days). The average interval between scans was 10.8 months (SD 7.2), with a median of 8.2 months (IQR 6.0–13.7). Based on the previously reported accuracy of CT analyses for assessing muscle and adipose tissue, a measurement error of 2% was assumed [28]. Changes between −2% and +2% were thus considered ‘maintenance of tissue’ and groups were defined with tissue loss (> 2% decrease per 100 days) or tissue gain/maintenance (any increase or ≤ 2% decrease) [29]. For practical reasons, the term ‘gain of tissue’ is used below to describe both maintenance and gain of tissue.

### 2.3 Peripheral Blood Lymphocyte Classification

Data on peripheral blood lymphocyte subsets were collected from 48 patients prior to treatment (see Table S5 for patient characteristics). Briefly, 100 μL of whole peripheral blood from each patient was added to a flow cytometry tube. The following antibodies were then added to the tube: CD56‐PE (cat. no. A07788, Beckman Coulter Inc.), CD16‐PE (cat. no. A07766, Beckman Coulter Inc.), CD4‐APC (cat. no. A94682, Beckman Coulter Inc.), CD45‐Krome Orange (cat. no. B36294, Beckman Coulter Inc.), CD8‐Pacific Blue (cat. no. A82791, Beckman Coulter Inc.), CD19‐PC5.5 (cat. no. A66328, Beckman Coulter Inc.) and CD3‐APC (cat. no. 665333, BD Biosciences). The mixture was vortexed and incubated at room temperature in the dark for 30 min. After incubation, 2 mL of lysing solution (FACS Lysing Solution, BD, 349202, prepared as a 1:9 working solution in PBS) was added, followed by centrifugation at 1500 rpm for 5 min. The supernatant was discarded, and the pellet was resuspended in an appropriate amount of PBS, centrifuged again, and the supernatant was discarded. Finally, 450 μL of PBS was added before flow cytometric analysis (Navios flow cytometer, Beckman Coulter Inc.). Lymphocytes were identified as CD45^+^ cells, and T cells were identified as CD3^+^ cells. Further gating was done to identify CD3^+^CD4^+^ or CD3^+^CD8^+^ T cells. NK cells were identified as CD3^−^CD56^+^ cells, and B cells were identified as CD3^−^CD19^+^ cells. Results from peripheral blood lymphocyte classification were treated as continuous independent variables.

### 2.4 Multiplex Immunofluorescence Staining

Formalin‐fixed and paraffin‐embedded (FFPE) tumour tissues from 24 lung cancer patients who received immunotherapy were collected during surgery (after diagnosis and before start of therapy), including 20 patients with stage IV lung cancer with oligometastasis. This study has been approved by the Ethics Committee of the Affiliated Cancer Hospital of Zhengzhou University & Henan Cancer Hospital. The slides were dewaxed, rehydrated, subjected to antigen retrieval, autofluorescence quenching and blocked. Subsequently, the slides were incubated with the respective primary antibodies (anti‐CD3 epsilon antibody [Abcam, ab237721]; rabbit recombinant multiclonal CD4 antibody [Abcam, ab288724]; CD8 monoclonal antibody [Proteintech, 66868‐1‐Ig], anti‐FOXP3 antibody [Abcam, ab191416], PD‐L1 monoclonal antibody [CST, #13684]), followed by secondary antibodies (goat anti‐rabbit IgG [Abcam, ab205718] and goat anti‐mouse IgG [Abcam, ab205719]). Signal amplification was achieved by incubating the samples with appropriate tyramide‐fluorophore conjugates in an amplification buffer according to the manufacturer's instructions (Wuhan Bioqiandu Technology). All samples were stained with DAPI (Wuhan Bioqiandu Technology, B0011). Finally, samples were scanned (3DHistech, Hungary) and analysed. At least three random 200× fields of view were selected from each slide for imaging and subsequent quantification using Image‐Pro Plus 6.0 software (Media Cybernetics Inc., Rockville, MD, USA). Briefly, cells marked with the corresponding fluorescent signals were selected as the standard for determining positive cells across all images. DAPI‐stained blue nuclei were used to identify the total number of cells. The percentage of positive cells (positive rate, %) was calculated by analysing each image using the ratio of positively stained cells to the total cell count. Finally, we determined the percentage of PD‐L1^+^ cells, CD4^+^ T cells, CD8^+^ T cells and FOXP3^+^ T regulatory (Treg) cells in the TILs. These percentages were treated as continuous independent variables.

### 2.5 Statistical Analysis

Data were analysed using IBM SPSS Statistics 27 for Microsoft Windows. Baseline characteristics and body composition parameters were grouped by treatment regimens (non‐immunotherapy cohort and immunotherapy cohort). Differences between baseline characteristics were tested using the two‐sided independent samples *t*‐test or Mann–Whitney *U* test for continuous variables and Fisher's exact test when assessing categorical variables. Univariable and multivariable logistic regression analysis were performed to assess the association between body composition parameters and OS. The hazard ratio (HR) for the associations between body composition parameters (SMI, VATI, SATI, SM‐RA, VAT‐RA, SAT‐RA), baseline clinical characteristics (sex, age, pathology, BMI), tumour‐related markers (PD‐L1 expression, NSE, CYFRA21‐1), inflammatory index (NLR), haematological parameters (NEU%, EOS%, BAS%, LYM%, MON%) and mortality were estimated using univariable and multivariable Cox regression analysis. Three models were used for Cox regression analysis: Model 1 represents the uncorrected univariable analysis; Model 2 represents a multivariable analysis including age, sex and relevant clinical characteristics selected by backward stepwise elimination (using a *p*‐value of < 0.1); and Model 3 represents a multivariable analysis including variables from Model 2 and body composition parameters selected by backward stepwise elimination (using a *p*‐value of < 0.1). Results are reported as HR and 95% confidence interval (CI). Variables with a *p*‐value < 0.10 in univariable analysis were subsequently included in the multivariable model. Time to censoring or event was indicated by the time in months from the cancer diagnosis date to the date of death or patients' last contact, respectively. OS was defined as the date of CT before chemotherapy until the date of death or last contact with the patient. Kaplan–Meier curves were plotted to estimate OS over time, and the log‐rank test was used to compare the difference in survival between groups.

Univariable and multivariable logistic regression analyses were used to explore the associations between peripheral blood lymphocyte subsets and baseline SMI or SMI changes. Given the natural influence of sex and age on SMI, these two variables were added to all models. The peripheral blood lymphocyte parameters analysed included CD3^+^, CD3^+^CD4^+^, CD3^+^CD8^+^ percentages and the CD4^+^/CD8^+^ ratio. Independent samples *t*‐tests were conducted to compare TIL levels between the SMI stable and SMI loss groups within the subset of 24 patients. Missing data were addressed using multiple imputation, generating *m* = 5 imputed datasets to ensure robustness of the results. A *p*‐value of < 0.05 was considered significant.

## 3 Results

### 3.1 Patient Characteristics

A total of 200 patients with advanced lung cancer receiving first‐line treatment were included in the study. Of these, 81 (40.5%) received immunotherapy (single treatment or in combination with other therapies; see Table S1 for patient characteristics) in the first line, and 119 (59.5%) received other therapies in the first line, but no immunotherapy.

The cohort comprised 128 males (64%) and 72 females (38%). The median (IQR) age of the total cohort was 60 (53, 66) years. We measured the body composition of all patients using a pretreatment CT scan. The median SMI at baseline of females and males were 40.7 and 51.0 cm^2^/m^2^, respectively, and these values were used as cut‐offs to define high and low baseline SMI in the study population. Table 1 shows the clinical characteristics and body composition parameters for the whole cohort and for the high‐ and low‐baseline SMI groups. Patients in the low‐SMI group had a significantly higher percentage of circulating mononuclear cells (5.4% vs. 4.9%; *p* = 0.005), CD3^+^ lymphocytes (68.5% vs. 64.9%; *p* = 0.027) and CD4^+^ T cells (40.2% vs. 34.7%; *p* = 0.009) compared with those in the high‐SMI group.

**TABLE 1:** Baseline clinical characteristics of 200 lung cancer patients.

	Whole	High SMI (*n* = 100)	Low SMI (*n* = 100)	*p*
Age (years)	60.0 (53.0, 66.0)	56.0 (51.3, 63.8)	62.0 (55.3, 67.0)	**0.005**
Sex M/F (%)	128/72	64/36	64/36	**1.000**
cTNM *n* (%)^a^				0.051
II	1	1	0	
III	50	23	27	
IV	145	74	71	
PD‐L1 *n* (%)				0.674
Negative	37	18	19	
Positive	55	24	31	
Unknown	108	58	50	
Regimen *n* (%)			100	0.773
Non‐immunotherapy	119	58	61	
Immunotherapy	81	42	39	
RECIST				0.480
No PD	96	45	51	
PD	104	55	49	
Tumour markers				
CEA^a^	4.9 (3.0, 18.1)	4.3 (3.0, 11.1)	3.2 (6.1, 29.6)	0.088
NSE^a^	18.4 (13.5, 33.4)	18.8 (13.8, 37.6)	18.0 (13.2, 30.2)	0.556
CYFRA21‐1^a^	4.7 (2.9, 9.5)	4.0 (2.6, 7.8)	5.0 (3.3, 11.9)	0.033
NLR				0.258
	97	53	44	
≥ 3	103	47	56	
Peripheral blood lymphocyte classification				
CD3^+^ (%)	68.0 (60.9, 72.8)	64.9 (56.7, 71.7)	68.5 (65.5, 74.8)	**0.027**
CD3^+^CD4^+^ (%)	38.1 (33.8, 42.9)	34.7 (30.7, 40.2)	40.2 (37.0, 43.4)	**0.009**
CD3^+^CD8^+^ (%)	24.8 (19.4, 30.1)	26.4 (20.2, 30.0)	24.0 (18.6, 30.3)	0.529
CD4^+^/CD8^+^	1.6 (1.2, 2.1)	1.4 (1.0, 2.2)	1.8 (1.3, 2.1)	0.164
B cell (%)	10.8 (7.5, 14.4)	10.8 (7.9, 14.4)	10.8 (7.0, 14.4)	0.910
NK cell (%)	18.8 (14.5, 25.4)	20.7 (14.6, 31.3)	16.7 (14.3, 22.1)	0.100
Haematological parameters				
WBC	7.3 (6.0, 8.8)	7.4 (6.3, 8.9)	6.7 (5.8, 8.8)	0.131
NEU%	68.6 (62.2, 74.6)	68.3 (61.1, 74.2)	70.0 (63.1, 74.9)	0.391
EOS%	1.6 (0.8, 3.3)	1.5 (0.8, 3.18)	1.8 (0.8, 3.7)	0.303
BAS%	0.4 (0.3, 0.6)	0.4 (0.3, 0.7)	0.4 (0.2, 0.6)	0.391
LYM%	22.8 (17.4, 28.5)	23.9 (18.4, 30.1)	21.4 (16.7, 27.4)	0.080
MON%	5.1 (4.2, 6.0)	4.9 (4.0, 5.7)	5.4 (4.4, 6.4)	**0.005**
RBC	4.5 (4.2, 4.9)	4.6 (4.3, 5.0)	4.4 (4.0, 4.8)	**< 0.001**
Hb (g/L)	136.0 (127.0, 148.0)	139.0 (129.3, 151.0)	134.0 (123.3, 145.0)	**0.011**
Body composition				
Weight (kg)	66.5 (59.0, 75.0)	70.0 (63.0, 77.5)	61.0 (55.0, 70.0)	**< 0.001**
BMI (kg/m^2^)	24.1 (21.5, 26.4)	25.7 (23.7, 27.3)	21.9 (20.3, 24.4)	**< 0.001**
SMI (cm^2^/m^2^)	46.6 (41.1, 52.8)	52.7 (46.6, 57.6)	41.3 (37.5, 46.7)	**< 0.001**
VATI (cm^2^/m^2^)	35.1 (14.9, 51.6)	44.7 (23.7, 60.9)	23.9 (11.7, 45.0)	**< 0.001**
SATI (cm^2^/m^2^)	41.1 (27.1, 64.0)	49.2 (32.9, 70.3)	37.4 (24.5, 54.5)	**< 0.001**
SM‐RA (HU)	37.1 (31.1, 42.6)	39.2 (32.3, 42.8)	35.7 (29.7, 42.2)	**0.046**
VAT‐RA (HU)	−90.4 (−95.7, −82.1)	−93.1 (−96.7, −83.3)	−88.2 (−94.5, −80.9)	**0.018**
SAT‐RA (HU)	−92.5 (−98.7, −84.4)	−93.2 (−98.5, −86.0)	−92.0 (−98.7, −82.5)	0.572

*Note:* The cut‐off for low‐ and high‐SMI groups was set by median of male and female SMI. An independent *t*‐test or Mann–Whitney *U* test was used for continuous variables. Fisher's exact test when comparing categorical variables. Data are presented as median (IQR).

Abbreviations: BAS%: basophil %; CEA: carcinoembryonic antigen; CYFRA21‐1: cytokeratin 19; EOS%: eosinophils %; Hb (g/L): haemoglobin; LYM%: lymphocyte %; MON%: monocyte %; NEU%: neutrophil %; NLR: neutrophil/lymphocyte ratio; NSE: neuron‐specific enolase; RBC: red blood cell; WBC: white blood cell.

^a^ Missing data: cTNM (*n* = 3), CEA (*n* = 16), NSE (*n* = 19), CYFRA21‐1 (*n* = 17).

According to the body composition changes after follow‐up, 192 patients were divided into SMI stable (any increase or < 2% decrease per 100 days) or SMI loss groups (defined by ≥ 2% decrease per 100 days). There was a significant difference in the proportion of baseline peripheral CD8^+^ T cells (CD3^+^CD8^+^) between the SMI stable and SMI loss groups (27.0% vs. 21.6%; *p* = 0.04) (Table 2).

**TABLE 2:** Clinical characteristics of 192 lung cancer patients according to change of SMI.

	Whole	SMI stable (*n* = 124)	SMI loss (*n* = 68)	*p*
Age (years)	60.0 (53.0, 66.0)	59.0 (52.3, 65.0)	61.5 (54.3, 66.0)	0.804
Sex M/F (%)	122/70	78/46	44/24	0.135
cTNM *n* (%)^a^				0.696
II	1	1	0	
III	47	29	18	
IV	140	91	49	
PD‐L1 *n* (%)				0.817
Negative	36	24	12	
Positive	54	38	16	
Unknown	102	62	40	
Regimen *n* (%)				0.520
Non‐ immunotherapy	116	77	39	
Immunotherapy	76	47	29	
RECIST				0.265
No PD	92	62	30	
PD	100	62	38	
Tumour markers				
CEA^a^	5.2 (3.1, 19.8)	4.3 (2.8, 18.6)	8.7 (3.6, 22.0)	0.074
NSE^a^	18.4 (13.6, 33.8)	18.4 (13.3, 32.9)	19.0 (13.9, 38.0)	0.431
CYFRA21‐1^a^	4.7 (2.8, 9.8)	4.1 (2.7, 9.0)	5.2 (3.5, 12.3)	0.125
NLR(3)				0.547
< 3	94	63	31	
≥ 3	98	61	37	
Peripheral blood lymphocyte classification				
CD3^+^ (%)	68.1 (61.4, 73.9)	68.2 (63.10 74.6)	68.0 (56.1, 71.8)	0.366
CD3^+^CD4^+^ (%)	38.0 (34.0, 42.9)	38.2 (34.3, 41.7)	36.5 (32.4, 43.4)	0.886
CD3^+^CD8^+^ (%)	25.0 (20.2, 29.9)	27.0 (23.1, 35.6)	21.6 (16.5, 28.0)	**0.040**
CD4^+^/CD8^+^	1.6 (1.2, 2.1)	1.4 (1.0, 1.9)	1.9 (1.2, 2.6)	0.105
B cell (%)	10.0 (7.1, 14.0)	10.0 (7.4, 13.4)	10.9 (6.6, 14.8)	0.547
NK cell (%)	17.5 (14.5, 24.8)	17.5 (14.4, 23.1)	18.4 (14.4, 30.6)	0.808
Haematological parameters				
WBC	7.2 (5.9, 8.9)	7.3 (5.9, 9.2)	6.8 (5.9, 8.1)	0.377
NEU%	68.5 (62.1, 74.8)	68.1 (62.7, 74.6)	70.9 (60.2, 75.1)	0.884
EOS%	1.7 (0.8, 3.3)	1.7 (0.9, 3.4)	1.4 (0.7, 3.3)	0.336
BAS%	0.4 (0.3, 0.6)	0.4 (0.3, 0.6)	0.4 (0.3, 0.7)	0.954
LYM%	22.8 (17.2, 28.7)	23.3 (17.0, 27.9)	22.3 (17.7, 30.0)	0.566
MON%	5.2 (4.2, 6.0)	5.2 (4.1, 6.0)	5.1 (4.3, 6.0)	0.821
RBC	4.5 (4.2, 4.9)	4.5 (4.2, 5.0)	4.5 (4.2, 4.8)	0.352
Hb (g/L)	136.0 (127.3, 148.0)	136.5 (127.0, 149.8)	134.5 (128.0, 147.3)	0.804
Body composition				
Weight (kg)	66.3 (59.0, 75.0)	66.3 (59.0, 75.0)	65.8 (57.1, 74.3)	0.452
BMI (kg/m^2^)	24.1 (21.5, 26.4)	24.1 (21.7, 26.3)	23.9 (20.9, 26.5)	0.441
SMI (cm^2^/m^2^)	46.1 (40.9, 52.7)	46.1 (40.7, 52.4)	46.0 (41.0, 53.9)	0.359
VATI (cm^2^/m^2^)	35.1 (14.9, 52.4)	35.5 (16.4, 56.6)	34.2 (13.5, 49.0)	0.301
SATI (cm^2^/m^2^)	41.6 (27.4, 64.0)	42.5 (28.2, 63.3)	38.1 (25.2, 66.3)	0.513
SM‐RA (HU)	37.0 (31.0, 42.6)	36.9 (31.1, 42.6)	37.0 (30.6, 42.8)	0.921
VAT‐RA (HU)	−90.4 (−95.8, −82.1)	−89.8 (−96.5, −83.1)	−91.4 (−94.8, −78.6)	0.260
SAT‐RA (HU)	−92.7 (−98.7, −85.1)	−93.0 (−98.8, −86.0)	−92.6 (−96.9, −81.1)	0.136

*Note:* The cut‐off for stable and loss of SMI groups was set by 2% decrease per 100 days. An independent *t*‐test or Mann–Whitney *U* test was used for continuous variables. Fisher's exact test when comparing categorical variables. Data are presented as median (IQR).

Abbreviations: BAS%: basophil %; CEA (ng/mL): carcinoembryonic antigen; CYFRA21‐1 (ng/mL): cytokeratin 19; EOS%: eosinophils %; Hb (g/L): haemoglobin; LYM%: lymphocyte %; MON%: monocyte %; NEU%: neutrophil %; NLR: neutrophil/lymphocyte ratio; NSE (ng/mL): neuron‐specific enolase; RBC (10^9/L): red blood cell; WBC (10^9/L): white blood cell.

^a^ Missing data: cTNM (*n* = 3), CEA (*n* = 12), NSE (*n* = 18), CYFRA21‐1 (*n* = 17).

### 3.2 Survival Analysis

**FIGURE 2:**
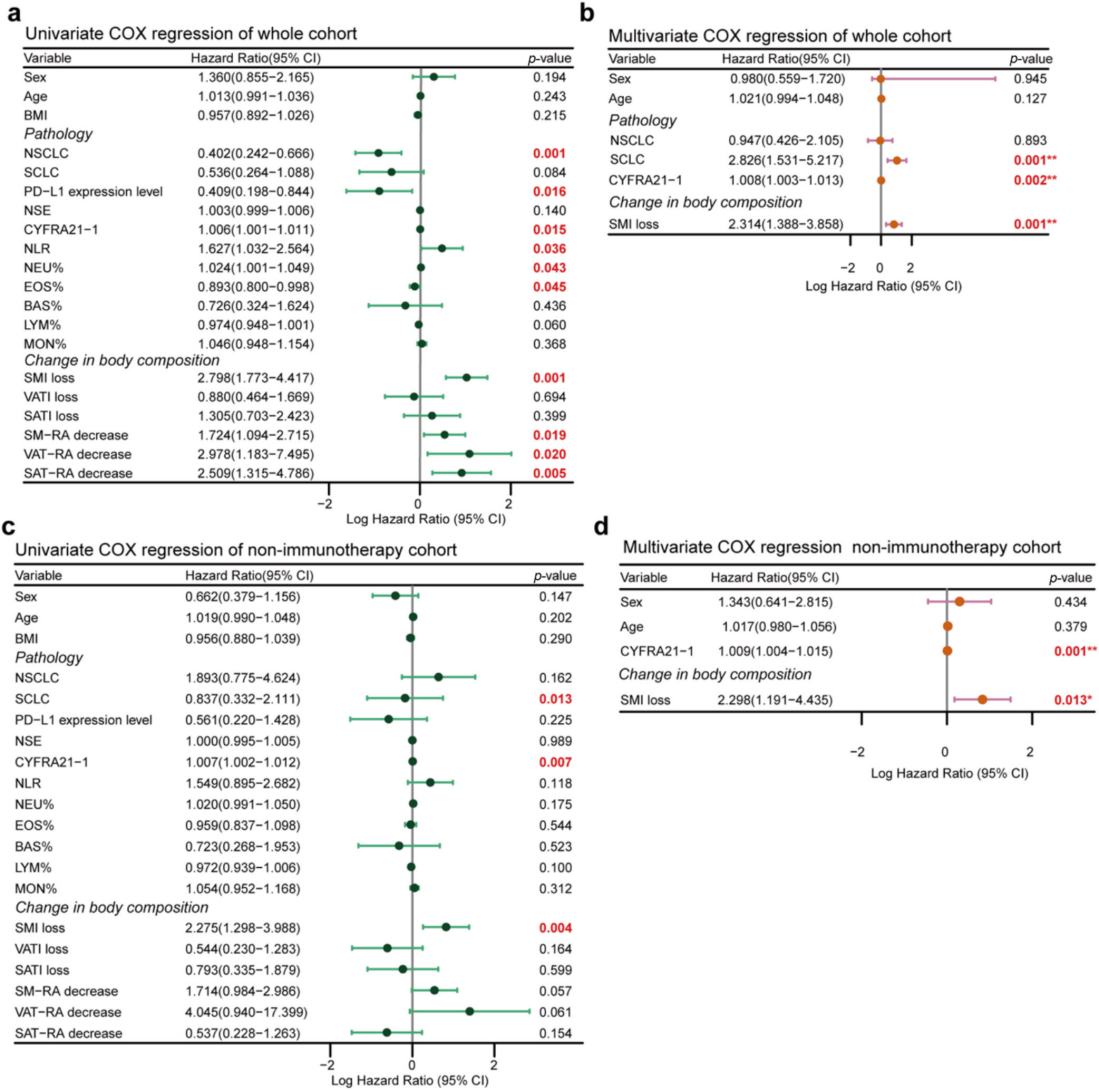
Cox proportional hazard models for the association of various parameters with OS in the whole and non‐immunotherapy cohort. (a and c) Univariate Cox regression analysis; (b and d) multivariate Cox regression analysis.

**FIGURE 3:**
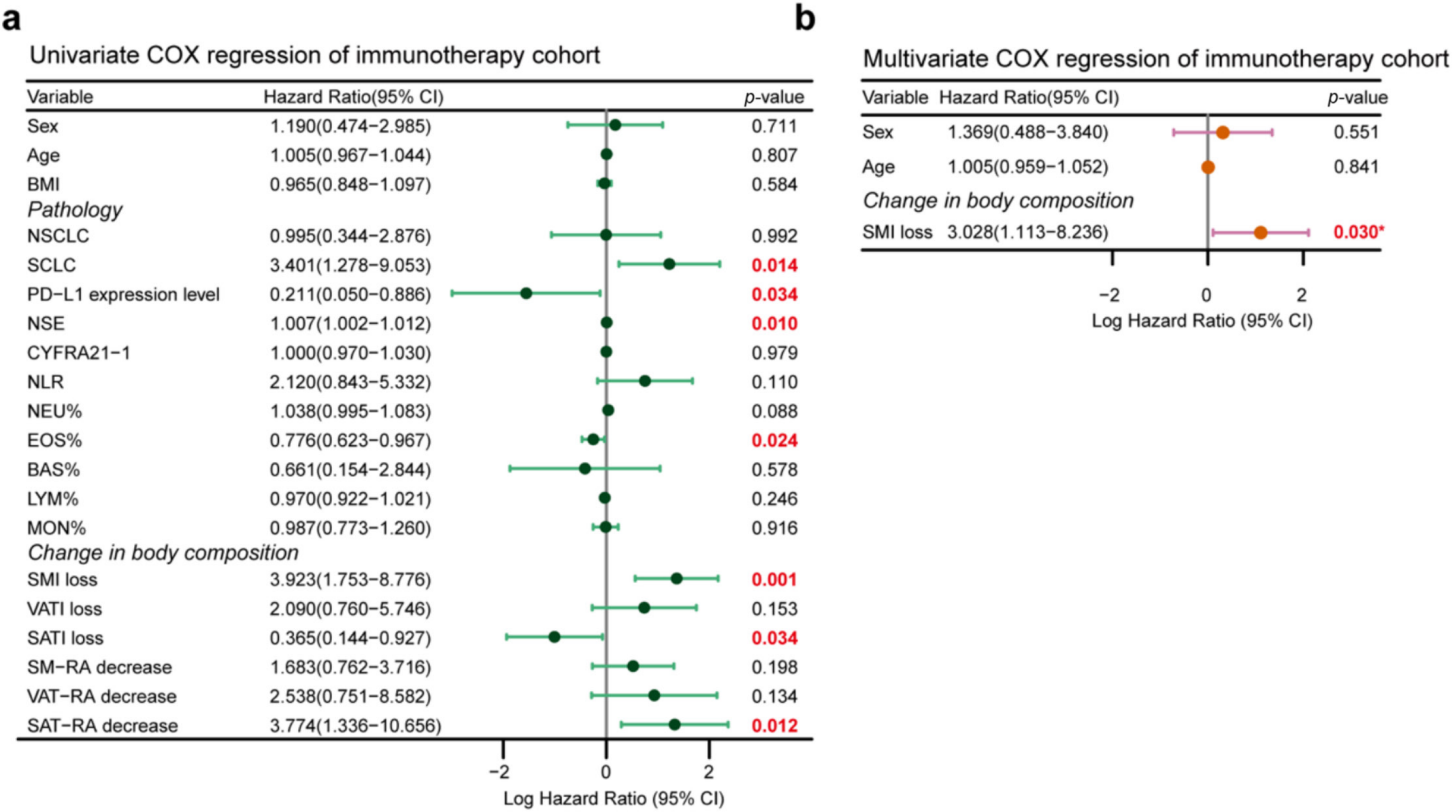
Cox proportional hazard models for the association of various parameters with OS in the immunotherapy cohort. (a) Univariate Cox regression analysis; (b) multivariate Cox regression analysis.

Results of univariable and multivariable Cox regression analysis are presented in Figures 2, 3. In multivariable analysis of the whole cohort, small cell lung cancer versus non‐small cell lung cancer (HR = 2.826, 95% CI = 1.531–5.217, *p* = 0.001), CYFRA21‐1 (HR = 1.008, 95% CI = 1.003–1.013, *p* = 0.002) and SMI loss (HR = 2.314, 95% CI = 1.388–3.858, *p* = 0.001) were significantly associated with OS (Figure 2b). Similarly, CYFRA21‐1 (HR = 1.009, 95% CI = 1.004–1.015, *p* = 0.001) and SMI loss (HR = 2.298, 95% CI = 1.191–4.435, *p* = 0.013) were significantly associated with OS in the nonimmunotherapy cohort (Figure 2d). Furthermore, only SMI loss (HR = 3.028, 95% CI = 1.113–8.236, *p* = 0.030) was independently associated with OS in the immunotherapy cohort (Figure 3b). Baseline body composition parameters were not associated with OS (Tables S2–S4).

**FIGURE 4:**
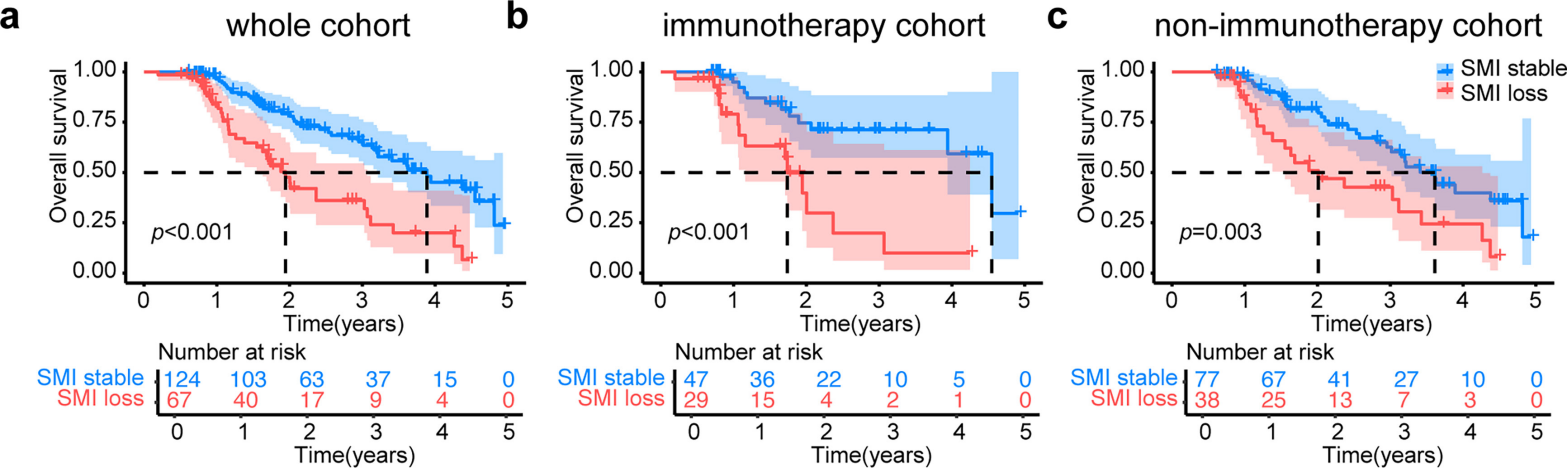
Prognostic differences among patients with stable SMI or SMI loss. The Kaplan–Meier curves represent the overall survival time in (a) the whole cohort, (b) the non‐immunotherapy cohort and (c) the immunotherapy cohort (one patient's survival data was unavailable, and eight patients were excluded due to unsuitable CT images).

Three Kaplan–Meier curves were generated to estimate survival probability based on the baseline SMI or SMI changes. To explore the relationship between SMI change and OS, patients were categorized into two groups: SMI stable and SMI loss. In the whole cohort, patients with SMI loss had shorter survival compared with patients with stable SMI (median survival: 1.165 vs. 2.030 years, *p* < 0.001) (Figure 4a). Similarly, in the immunotherapy cohort, patients with SMI loss showed lower survival compared with patients with stable SMI (median survival, 1.061 vs. 1.767 years, *p* < 0.001) (Figure 4b). In the no‐immunotherapy cohort, patients with SMI loss also had shorter survival (Figure 4c) (median survival, 1.381 vs. 2.096 years, *p* = 0.003). The OS of patients with high baseline SMI versus patients with low baseline SMI was not different in any cohort (Figure S3). Patients with stable SMI and positive PD‐L1 expression exhibited the greatest survival benefit across all cohorts (Figure S4).

### 3.3 Circulating Immune Cells and SM Mass

**FIGURE 5:**
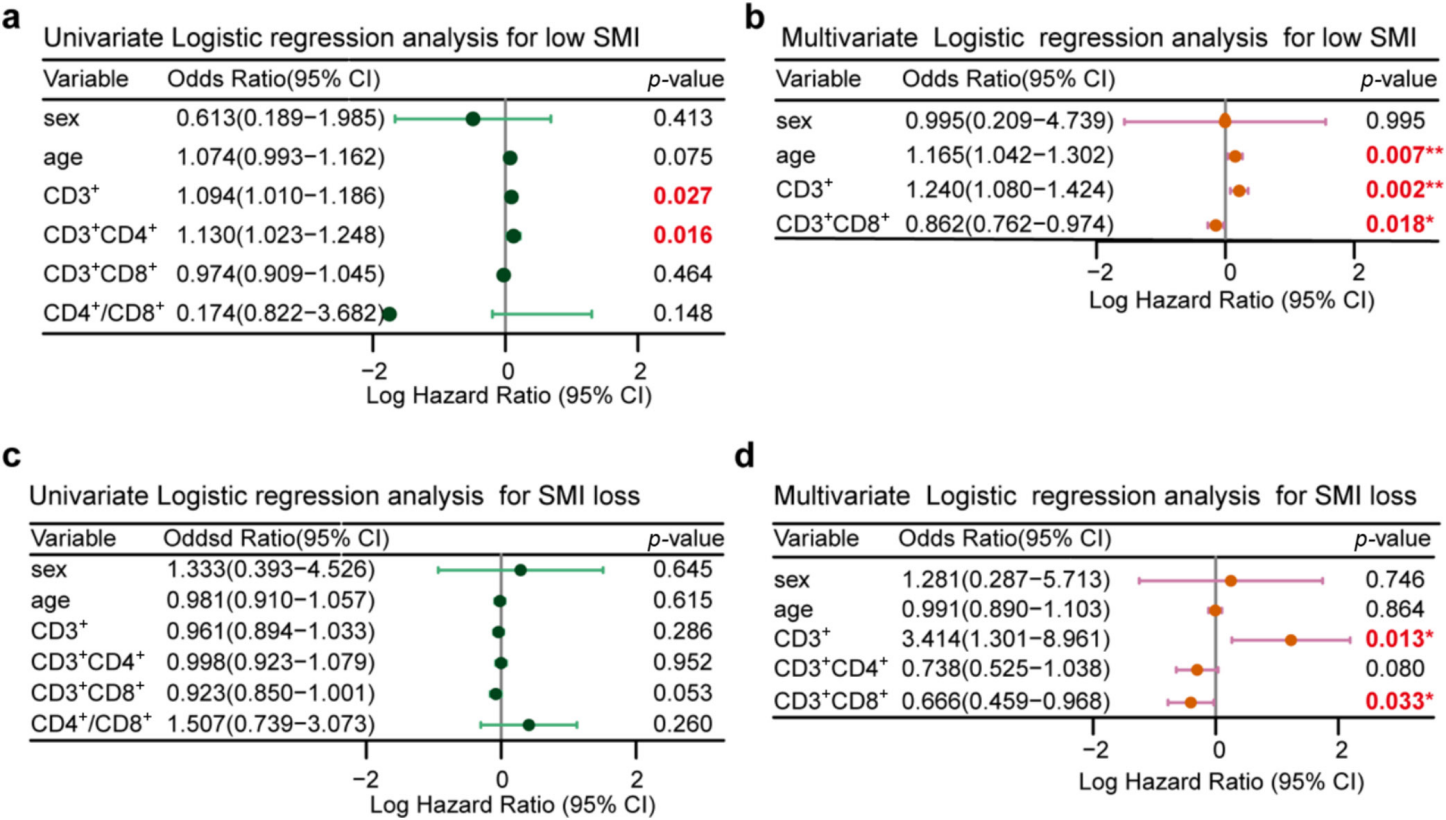
Results of logistic regression analysis for low SMI and SMI loss. Univariate (a) and multivariate (b) logistic regression analysis results for low SMI. Univariate (c) and multivariate (d) logistic regression analysis results for SMI loss.

As shown in Figure 5, higher age (OR = 1.165, 95% CI = 1.042–1.302, *p* = 0.007), higher CD3^+^ T cell proportion (OR = 1.240, 95% CI = 1.080–1.424, *p* = 0.002) and lower CD3^+^CD8^+^ T cell proportion (OR = 0.862, 95% CI = 0.762–0.974, *p* = 0.018) were associated with low SMI in multivariable analysis (Figure 5b). Furthermore, higher CD3^+^ T cell proportion (OR = 3.414, 95% CI = 1.301–8.961, *p* = 0.013) and lower CD3^+^CD8^+^ T cell proportion (OR = 0.666, 95% CI = 0.459–0.968, *p* = 0.033) were associated with posttreatment SMI loss, and lower CD4^+^ T cell numbers (OR = 0.738, 95% CI = 0.525–1.038, *p* = 0.080) tended to be associated with this factor (Figure 5d).

### 3.4 TILs and SM Mass

**FIGURE 6:**
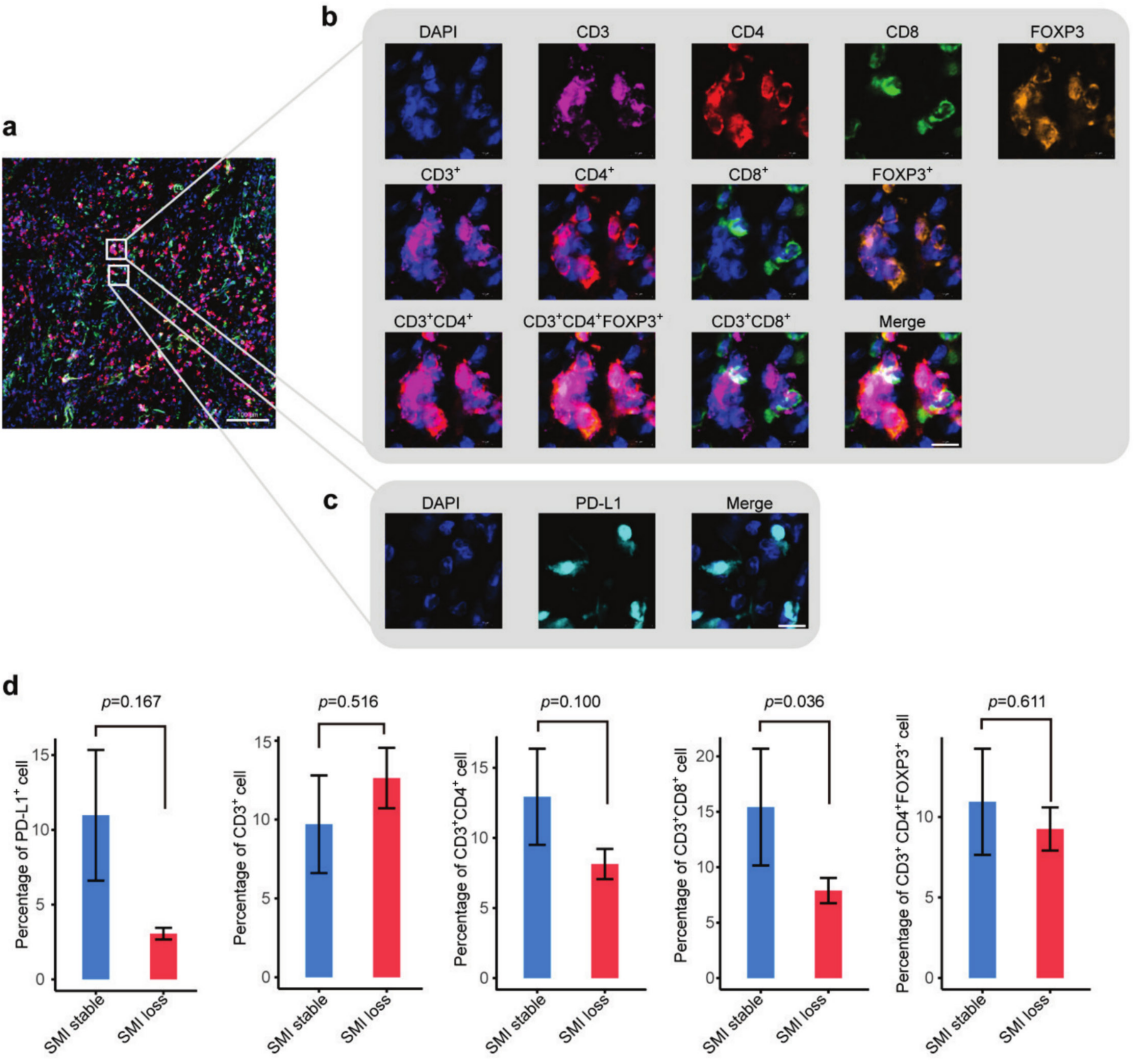
Localization and abundance of specific immune cell types within the tumour and their relationship with skeletal muscle loss during therapy. (a) Representative immunofluorescence image of merged stainings (bar, 100 μm). (b–c) Representative immunofluorescence staining images showing the different cell populations identified (bar, 10 μm). (d) The percentages of cells in the tumour microenvironment expressing the indicated markers in patients with stable SMI versus SMI loss during therapy.

To further investigate the relationship between the tumour immune microenvironment and SMI loss in patients' posttreatment, we collected FFPE samples from 24 patients (see Table S6 for patient characteristics) and performed multiplex immunofluorescence staining to explore the correlation between TILs and changes in body composition following immunotherapy. Although no significant differences in the percentages of PD‐L1^+^ cells (11.0% ± 4.4% vs. 3.1% ± 0.4%, *p* = 0.167), CD3^+^ T cells (9.7% ± 3.1% vs. 12.6% ± 1.9%, *p* = 0.516), CD4^+^ T cells (12.9% ± 3.4% vs. 8.1% ± 1.1%, *p* = 0.100) or CD4^+^FOXP3^+^ cells (10.9% ± 3.3% vs. 9.3% ± 1.3%, *p* = 0.611) between the stable SMI and SMI loss groups were observed (Figure 6d), patients with stable SMI posttreatment exhibited a significantly higher number of CD8^+^ TILs compared with patients with SMI loss (15.4% ± 5.3% vs. 7.9% ± 1.1%, *p* = 0.036; Figure 6d). The difference in the percentage of TILs between the baseline highSMI and lowSMI groups was not significant (Figure S5).

## 4 Discussion

In the current study, we found that SM loss was associated with shorter survival of patients receiving systemic therapy for advanced lung cancer. Low baseline SM mass and loss of muscle mass during therapy were associated with the number of circulating CD3^+^ T cells and CD3^+^CD8^+^ T cells, and patients with SM loss had a lower number of CD8^+^ TILs than patients with stable muscle mass. It is important to note that our analyses of TIL and peripheral blood lymphocyte subsets were exploratory in nature and conducted in relatively small subgroups. Nevertheless, the observed associations between SM parameters and immune cell populations warrant further investigation.

SM loss in cancer patients receiving chemotherapy [19, 30, 31, 32] or immunotherapy [33] has previously been shown to be associated with poor clinical outcomes in several types of cancer, similar to what we observed in our study for both the chemotherapy and the immunotherapy cohort. Expanding on these data, our exploratory analysis of the relationship between SM status and immune cells revealed that the abundance of certain circulating immune cell populations appears to be associated with the SM state. Specifically, higher peripheral CD3^+^ T cell numbers and lower circulating CD8^+^ T cell numbers were identified to be associated with SMI loss during therapy. Low SMI before the start of therapy was also associated with these same peripheral T cell populations. To our knowledge, only a few studies have previously demonstrated a correlation between baseline body composition and circulating T cells. In a small pilot study of patients with gastrointestinal cancer [24], a lower proportion of circulating CD3^+^CD4^+^CD25^high^CD127^low^ regulatory T cells was found to be associated with a higher lean mass index. In that study, different CD8^+^ subsets were also shown to be correlated either negatively or positively with handgrip strength. More recently, Feng et al. showed that low SMI was associated with a reduced peripheral CD4^+^/CD8^+^ ratio in patients with NSCLC [16].

Of note, neither of these studies investigated the relationship between tumour‐infiltrating T cells and body composition. In our small TIL cohort (*n* = 24), we found that loss of SM during therapy was not only associated with the abundance of peripheral CD8^+^ cells but also characterized by a lower number of CD8^+^ cells in the tumour. Similar results were found in earlier studies focusing on the relationship between baseline body composition and tumour‐infiltrated lymphocytes. For example, low CD8^+^ TILs were associated with low pretreatment SMI or sarcopenia in patients with early‐stage pancreatic ductal adenocarcinoma [34], hepatocellular carcinoma [35], extrahepatic cholangiocarcinoma [36] or colorectal cancer [37]. Conversely, research utilizing rectus abdominis samples from gastrointestinal cancer patients demonstrated a positive correlation between the proportion of muscle‐infiltrating CD8^+^ T cells and muscle mass before surgery [25].

The underlying mechanisms governing the relationships between body composition and immune cells, and in particular the link between CD3^+^ and CD8^+^ T cells and muscle mass, remain undefined. Earlier explorations suggested that circulating CD8^+^ T cells may affect SM by influencing key molecules (e.g., ACVR1B/2B) involved in catabolic pathways [25]. Experimental models of cancer‐associated cachexia have shown that increased CD8^+^ T cells in muscle negatively correlate with cachexia [38], but no information on T cell abundance in the tumour or circulation was reported. Our preliminary observation that the abundance of CD8^+^ cells in the tumour was negatively correlated with SM loss during therapy suggests, but does not prove, that a strong CD8^+^ T cell antitumour response may protect against muscle catabolism by reducing tumour burden and consequently diminishing tumour‐derived catabolic signals. The positive association between the number of circulating CD3^+^ T cells and low pretreatment SMI, as well as loss of SMI during treatment, may be associated with an increased metabolic burden on the body because of a non‐specific increase in overall immune activity [39]. However, additional mechanistic studies are needed to confirm these hypotheses.

Our results highlight the importance of dynamic assessment of body composition changes during treatment to evaluate prognosis. When SMI change during treatment was included in the analysis, often‐used markers such as NLR, SII and PD‐L1 expression contributed minimally and non‐significantly to survival prediction. In contrast, SMI loss during treatment was the only factor that consistently exhibited independent prognostic value across all study cohorts. Notably, in the immunotherapy cohort, SMI loss was the sole marker with statistically significant prognostic capability for immunotherapy outcomes. This raises interest about the crosstalk between body composition evolution and tumour immune microenvironments during ICI therapies. As a crucial component of the tumour immune microenvironment, TILs are known to be closely associated with the therapeutic response to ICIs [21, 40]. Since we found a consistent association between intratumoural and peripheral CD8^+^ T cell abundance and SM loss, measurements of circulating immune cells may provide us with clues to explore the relationship between tumour lymphocytes, cachexia and immunotherapy response in cancer patients longitudinally in more detail. However, these associations should be interpreted cautiously, given the exploratory nature of our immune analyses.

Our findings support that SM loss quantified by routine CT scan analysis may help identify lung cancer patients at higher risk of poor outcomes during ICI therapy. Early recognition of low or declining SMI may allow for timely, multimodal interventions to help preserve muscle mass and potentially sustain the CD8^+^ T cell‐mediated antitumour immune response. Further prospective studies integrating longitudinal body composition assessment with immune profiling are warranted to assess whether muscle‐preservation strategies can enhance immunotherapy efficacy.

This study has several limitations. Firstly, the sample size of the immunotherapy cohort was relatively small and needs to be expanded to increase statistical power and generalizability of our findings. The sample sizes for our immune analyses were particularly limited (*n* = 24 for TILs and *n* = 48 for peripheral blood lymphocytes), restricting our ability to draw definitive conclusions about immune‐muscle interactions. Furthermore, future studies should consider collecting peripheral blood lymphocyte subsets pretreatment and posttreatment to further explore the impact of longitudinal changes. Such future studies should also be multi‐centre and pan‐cancer, ideally with patients with different stages of disease, to validate and generalize our findings across diverse populations and cancer types. Additionally, the molecular mechanisms by which CD8^+^ and CD3^+^ T cells may influence treatment‐related SMI changes have not been studied here, and causality has not been established. It should be explored in future studies if a more detailed analysis of inflammatory and metabolic markers such as IL‐6 and glucose might further improve the independent predictive value of body composition features for survival outcomes. Simultaneous investigation of immune cell abundances in blood, tumour and muscle, complemented with functional muscle measurements, may shed light on the robustness of the observed correlations and the potential mechanistic links. Meanwhile, because of the retrospective nature of the study, data on physical function and activity were unavailable for analysis. Future studies could prospectively evaluate changes in physical function and indicate if these changes coevolve with changes in body composition. Although patients with SMI loss showed lower RBC and haemoglobin levels, the nutritional risk scores (NRS 1–2) were relatively uniform, suggesting that malnutrition did not play a role. Further, we did not record prebaseline weight loss. Future prospective studies incorporating prediagnosis weight trajectories could help to more precisely distinguish pre‐existing versus treatment‐related muscle loss. However, weight history is highly subjective and should ideally be objectified (e.g., with recorded data of smart‐weight scales).

Additionally, we recognize the possible importance of sarcopenic obesity. However, our data show that dynamic changes in SMI develop independently from BMI. This suggests that changes in SM mass may reflect deeper tumour–host interactions rather than simple indicators of nutritional status.

## 5 Conclusion

Our study explored for the first time the relationship between body composition changes during first‐line treatment and prognosis of patients with advanced lung cancer. Patients who developed SMI loss during treatment showed significantly poorer OS. SMI loss was an independent predictor of poor prognosis, both in patients treated without immunotherapy and in patients treated with immunotherapy. Our exploratory analyses suggest that higher relative abundance of peripheral CD3^+^ cells and lower relative abundance of CD8^+^ cells were associated with SMI loss, and a low proportion of CD8^+^ TIL was protective against SMI loss during treatment of advanced lung cancer patients receiving immunotherapy. These findings warrant validation in larger prospective studies.

## Ethics Statement

This study has been approved by the Ethics Committee of the Affiliated Cancer Hospital of Zhengzhou University & Henan Cancer Hospital and has therefore been performed in accordance with the ethical standards laid down in the 1964 Declaration of Helsinki and its later amendments.

## Conflicts of Interest

The authors declare no conflicts of interest.

## Supporting information


**Table S1** Baseline clinical characteristics of 200 lung cancer patients.
**Table S2** Cox proportional hazard models for the association of various parameters with OS in the whole cohort.
**Table S3** Cox proportional hazard models for the association of various parameters with OS in the immunotherapy cohort.
**Table S4** Cox proportional hazard models for the association of various parameters with OS in the non‐immunotherapy cohort.
**Table S5** Characteristics of 48 lung cancer patients analysed for peripheral blood lymphocyte abundance.
**Table S6** Characteristics of 24 lung cancer patients analysed for tumour immune cell infiltration.
**Table S7** Nutrition risk screening form for adult inpatients.
**Figure S1** Cox proportional hazard models for the association of various parameters with OS in the whole and non‐immunotherapy cohort. (a and c) Univariate Cox regression analysis; (b and d) multivariate Cox regression analysis.
**Figure S2** Cox proportional hazard models for the association of various parameters with OS in the immunotherapy cohort. (a) Univariate Cox regression analysis; (b) multivariate Cox regression analysis.
**Figure S3** Prognostic differences among patients with different high SMI and low SMI. The Kaplan–Meier curves represent the overall survival time in (a) whole cohort, (b) immunotherapy cohort and (c) non‐immunotherapy cohort (one patient's survival data was unavailable).
**Figure S4** Survival analysis according to baseline SMI or SMI change during therapy combined with PD‐L1 expression status. Kaplan–Meier curves of baseline SMI combined with PD‐L1 expression status in the whole cohort (a), immunotherapy cohort (b) and no‐immunotherapy cohort (c). Kaplan–Meier curves of SMI change combined with PD‐L1 expression status in the whole cohort (d), immunotherapy cohort (e) and non‐immunotherapy cohort (f).
**Figure S5** The percentages of different TILs in patients with low versus high pretreatment skeletal muscle mass. The percentages of cells in the tumour microenvironment expressing the indicated markers are shown for patients with low versus high SMI at baseline.
