# Supplementary material for: Loss of Skeletal Muscle Mass Is Associated With Reduced Cytotoxic T Cell Abundance and Poor Survival in Advanced Lung Cancer
Source: J Cachexia Sarcopenia Muscle. 2025 Sep 10;16(5):e70063. doi: 10.1002/jcsm.70063 (PMC12422944; doi:10.1002/jcsm.70063)
Supplement: Supplementary file 1 — Table S1 Baseline clinical characteristics of 200 lung cancer patients. Table S2 Cox proportional hazard models for the association of various parameters with OS in the whole cohort. Table S3 Cox proportional hazard models for the association of various parameters with OS in the immunotherapy cohort. Table S4 Cox proportional hazard models for the association of various parameters with OS in the non‐immunotherapy cohort. Table S5 Characteristics of 48 lung cancer patients analysed for peripheral blood lymphocyte abundance. Table S6 Characteristics of 24 lung cancer patients analysed for tumour immune cell infiltration. Table S7 Nutrition risk screening form for adult inpatients. Figure S1 Cox proportional hazard models for the association of various parameters with OS in the whole and non‐immunotherapy cohort. (a and c) Univariate Cox regression analysis; (b and d) multivariate Cox regression analysis. Figure S2 Cox proportional hazard models for the association of various parameters with OS in the immunotherapy cohort. (a) Univariate Cox regression analysis; (b) multivariate Cox regression analysis. Figure S3 Prognostic differences among patients with different high SMI and low SMI. The Kaplan–Meier curves represent the overall survival time in (a) whole cohort, (b) immunotherapy cohort and (c) non‐immunotherapy cohort (one patient's survival data was unavailable). Figure S4 Survival analysis according to baseline SMI or SMI change during therapy combined with PD‐L1 expression status. Kaplan–Meier curves of baseline SMI combined with PD‐L1 expression status in the whole cohort (a), immunotherapy cohort (b) and no‐immunotherapy cohort (c). Kaplan–Meier curves of SMI change combined with PD‐L1 expression status in the whole cohort (d), immunotherapy cohort (e) and non‐immunotherapy cohort (f). Figure S5 The percentages of different TILs in patients with low versus high pretreatment skeletal muscle mass. The percentages of cells in the tumour microenvironment expressi [file JCSM-16-e70063-s001.docx]

| **Table S1. Baseline clinical characteristics of 200 lung cancer patients** | | | | |
| --- | --- | --- | --- | --- |
|  | Whole cohort | Non-immunotherapy cohort | Immunotherapy cohort | *p*-value |
| Age | 60.0 (53.0, 66.0) | 60.0 (53.0, 65.0) | 61.0 (54.0, 67.0) | 0.452 |
| Gender M/F (%) | 128/72 (64.0%) | 65/54 (54.6%) | 63/18 (77.8%) | <0.001 |
| Weight (Kg) | 66.5 (59.0, 75.0) | 65.0 (57.0, 75.0) | 68.0 (60.0, 75.0) | 0.137 |
| BMI (Kg/m^2^) | 24.1 (21.5, 26.4) | 24.0 (21.5, 26.5) | 24.1 (21.5, 26.2) | 0.872 |
| Cancer subtype |  |  |  | 1.000 |
| NSCLC | 150 (75.0%) | 89 (74.8%) | 61 (75.3%) |  |
| SCLC | 50 (25.0%) | 30 (25.2%) | 20 (24.7%) |  |
| cTNM n (%) |  |  |  | 0.433 |
| II | 1 (0.5%) | 0 (0%) | 1 (1.2%) |  |
| III | 50 (25.0%) | 28 (23.5%) | 22 (27.2%) |  |
| IV | 145 (72.5%) | 87 (73.1%) | 58 (71.6%) |  |
| Metastasis pattern |  |  |  |  |
| brain | 30 (15.4%) | 19 (16.4%) | 11 (13.9%) | 0.548 |
| pleura | 30 (15.4%) | 20 (17.2%) | 10 (12.7%) |  |
| bone | 27 (13.8%) | 19 (16.4%) | 8 (10.1%) |  |
| brain and bone | 18 (9.2%) | 10 (8.6%) | 8 (10.1%) |  |
| lungs | 14 (7.2%) | 5 (4.3%) | 9 (11.4%) |  |
| liver | 13 (6.7%) | 7 (6%) | 6 (7.6%) |  |
| adrenal gland | 8 (4.1%) | 4 (3.4%) | 4 (5.1%) |  |
| others | 5 (2.6%) | 4 (3.4%) | 1 (1.3%) |  |
| PD-L1 n (%) |  |  |  | 0.115 |
| Negative | 37 (18.5%) | 25 (21.0%) | 12 (14.8%) |  |
| Positive | 55 (27.5%) | 28 (23.5%) | 27 (33.3%) |  |
| Unknown | 108 (54.0%) | 66 (55.4%) | 48 (59.2%) |  |
| RECIST |  |  |  | 0.933 |
| no PD | 97 (48.5%) | 58 (48.7%) | 39 (48.1%) |  |
| PD | 103 (51.5%) | 61 (51.3%) | 42 (51.9%) |  |
| CEA | 4.9 (3.0, 18.1) | 7.6 (2.8, 23.1) | 4.3 (3.2, 9.9) | 0.101 |
| NSE (ng/mL) | 18.4 (13.5, 33.4) | 18.2 (13.8, 29.9) | 19.0 (13.2, 38.0) | 0.715 |
| CYFRA21-1(ng/ml) | 4.7 (2.9, 9.5) | 4.3 (2.6, 9.1) | 5.0 (3.3, 10.9) | 0.105 |
| NLR (3) |  |  |  | 0.250 |
| <3 | 97 (48.5%) | 62 (52.1%) | 35 (43.2%) |  |
| ≥3 | 103 (51.5%) | 57 (47.9%) | 46 (56.8%) |  |
| CRP | 4.5 (1.9, 22.5) | 4.2 (1.2, 15.1) | 11.5 (2.5, 29.5) | 0.017 |
| NEU% | 68.6 (62.2, 74.6) | 68.4 (61.6, 74.3) | 68.7 (64.1, 75.4) | 0.344 |
| EOS% | 1.6 (0.8, 3.3) | 1.5 (0.8,3.2) | 1.8 (0.9, 3.8) | 0.260 |
| BAS% | 0.4 (0.3, 0.6) | 0.4 (0.3, 0.7) | 0.4 (0.25, 0.6) | 0.078 |
| LYM% | 22.8 (17.4, 28.5) | 23.6 (17.9, 28.9) | 21.3 (16.4, 27.2) | 0.121 |
| MON% | 5.1 (4.2, 6.0) | 5.1 (4.2, 6.0) | 5.1 (4.3, 6.1) | 0.854 |
| SMI (cm^2^/m^2^) | 46.6 (41.1, 52.8) | 44.6 (39.7, 52.3) | 48.0 (42.2, 53.2) | 0.030 |
| VATI (cm^2^/m^2^) | 35.1 (14.9, 51.6) | 37.5 (15.1, 51.2) | 33.3 (14.4, 53.8) | 0.867 |
| SATI (cm^2^/m^2^) | 41.1 (27.1, 64.0) | 43.5 (28.9, 65.7) | 37.0 (24.5, 60.5) | 0.075 |
| SM-RA (HU) | 37.1 (31.1, 42.6) | 37.1 (31.1, 43.1) | 37.0 (30.9, 42.1) | 0.478 |
| VAT-RA (HU) | -90.4 (-95.7, -82.1) | -90.5 (-95.8, -82.9) | -89.3 (-95.7, -79.8) | 0.585 |
| SAT-RA (HU) | -92.5 (-98.7, -84.4) | -93.2 (-98.4, -79.3) | -89.7 (-98.4, -79.3) | 0.096 |
| *Treatment* |  |  |  |  |
| Total | - | 119 (100) | 81 (100) |  |
| Immunotherapy | - | - | 2 (2.5%) | - |
| Immuno+chemotherapy | - | - | 44 (54.3%) | - |
| Immuno+targeted therapy | - | - | 4 (4.9%) | - |
| Immuno+chemo+radiotherapy | - | - | 13 (16.1%) | - |
| Immuno+chemo+targeted therapy | - | - | 18 (22.2%) | - |
| Chemotherapy | - | 21 (17.6%) | - | - |
| Targeted therapy | - | 30 (25.2%) | - | - |
| Chemo+radiotherapy | - | 20 (16.8%) | - | - |
| Chemo+targeted therapy | - | 41 (34.5%) | - | - |
| Radio+targeted therapy | - | 3 (2.5%) | - | - |
| Chemo+radio+targeted therapy | - | 4 (3.4%) | - | - |

| **Table S2. Cox proportional Hazard Models for the Association of various parameters with OS in the whole cohort** | | | | | |
| --- | --- | --- | --- | --- | --- |
|  | Univariate | *p*-value | Multivariate | *p*-value | |
| Sex | 1.416 (0.893, 2.245) | 0.139 | 2.642 (0.826, 8.452) | 0.101 | |
| Age | 1.012 (0.990, 1.035) | 0.275 | 1.036 (0.980, 1.096) | 0.213 | |
| Pathology |  | <0.001 |  |  | |
| NSCLC | 1.280 (0.651, 2.519) | 0.474 |  |  | |
| SCLC | 2.626 (1.618, 4.361) | <0.001 |  |  | |
| PD-L1 expression level | 0.429 (0.208, 0.885) | 0.022 | 0.117 (0.041, 0.335) | <0.001 | |
| NLR (3) | 1.594 (1.017, 2.497) | 0.042 | 2.275 (0.909, 5.692) | 0.079 | |
| NEU% | 1.025 (1.002, 1.049) | 0.035 |  |  | |
| EOS% | 0.896 (0.803, 0.999) | 0.048 |  |  | |
| BAS% | 0.694 (0.310, 1.555) | 0.375 |  |  | |
| LYM% | 0.974 (0.948, 1.000) | 0.052 |  |  | |
| MON% | 1.039 (0.941, 1.148) | 0.444 |  |  | |
| *Baseline body composition* | | | | |  |
| SMI (cm^2^/m^2^) | 0.904 (0.582, 1.406) | 0.655 | 0.330 (0.119, 0.916) | 0.033 | |
| VATI (cm^2^/m^2^) | 0.954 (0.615, 1.481) | 0.834 |  |  | |
| SATI (cm^2^/m^2^) | 1.018 (0.656, 1.580) | 0.935 | 3.124 (1.167, 8.362) | 0.023 | |
| SM-RA (HU) | 0.986 (0.635, 1.531) | 0.950 |  |  | |
| VAT-RA (HU) | 0.842 (0.542, 1.306) | 0.442 |  |  | |
| SAT-RA (HU) | 1.004 (0.647, 1.558) | 0.984 |  |  | |

| **Table S3. Cox proportional Hazard Models for the Association of various parameters with OS in the immunotherapy cohort** | | | | |
| --- | --- | --- | --- | --- |
|  | Univariate | *p*-value | Multivariate | *p*-value |
| Sex | 1.181 (0.473, 2.948) | 0.722 | 1.224 (0.421, 3.555) | 0.710 |
| Age | 1.003 (0.966, 1.042) | 0.881 | 0.983 (0.939, 1.030) | 0.477 |
| Pathology |  | 0.017 |  | 0.025 |
| NSCLC | 0.287 (0.110, 0.747) | 0.011 | 1.355 (0.412, 4.455) | 0.617 |
| SCLC | 0.258 (0.083, 0.799) | 0.019 | 3.969 (1.398, 11.273) | 0.010 |
| CEA | 1.000 (0.995, 1.006) | 0.939 |  |  |
| NSE (ng/mL) | 1.007 (1.002, 1.012) | 0.008 |  |  |
| CYFRA21-1 (ng/ml) | 0.999 (0.970, 1.030) | 0.952 |  |  |
| NLR (3) | 1.791 (0.749, 4.281) | 0.190 |  |  |
| CRP (2) | 0989 (0.970, 1.009) | 0.273 |  |  |
| NEU% | 1.036 (0.993, 1.080) | 0.098 | 1.087 (1.024, 1.155) | 0.006 |
| EOS% | 0.793 (0.642, 0.980) | 0.031 |  |  |
| BAS% | 0.596 (0.135, 2.637) | 0.496 |  |  |
| LYM% | 0.973 (0.925, 1.023) | 0.281 |  |  |
| MON% | 0.954 (0.751, 1.211) | 0.696 |  |  |
| *Baseline body composition* | | | | |
| SMI (cm^2^/m^2^) | 0.827 (0.381, 1.793) | 0.630 | 0.338 (0.131, 0.873) | 0.025 |
| VATI (cm^2^/m^2^) | 1.245 (0.576, 2.693) | 0.578 |  |  |
| SATI (cm^2^/m^2^) | 1.103 (0.518, 2.350) | 0.799 |  |  |
| SM-RA (HU) | 1.027 (0.476, 2.219) | 0.945 |  |  |
| VAT-RA (HU) | 0.634 (0.291, 1.382) | 0.252 |  |  |
| SAT-RA (HU) | 0.768 (0.361, 1.636) | 0.494 |  |  |

| **Table S4. Cox proportional Hazard Models for the Association of various parameters with OS in the non-immunotherapy cohort** | | | | | |
| --- | --- | --- | --- | --- | --- |
|  | Univariate | *p*-value | Multivariate | *p*-value | |
|  |  |  |  |  | |
| Sex | 1.598 (0.919, 2.777) | 0.097 | 0.804 (0.385, 1.679) | 0.592 | |
| Age | 1.018 (0.990, 1.047) | 0.215 | 1.013 (0.978, 1.049) | 0.483 | |
| Patholopy |  | 0.017 |  |  | |
| NSCLC | 0.435 (0.241, 0.786) | 0.006 |  |  | |
| SCLC | 0.837 (0.332, 2.111) | 0.706 |  |  | |
| PD-L1 expression level | 0.593 (0.233, 1.509) | 0.272 |  |  | |
| CEA | 1.000 (0.998, 1.002) | 0.943 |  |  | |
| NSE (ng/mL) | 1.000 (0.995, 1.005) | 0.965 |  |  | |
| CYFRA21-1 (ng/ml) | 1.007 (1.002, 1.011) | 0.007 | 1.009 (1.003, 1.014) | 0.001 | |
| NLR (3) | 0.624 (0.362, 1.075) | 0.089 |  |  | |
| CRP (2) | 1.011 (1.001, 1.022) | 0.035 |  |  | |
| NEU% | 1.022 (0.993, 1.052) | 0.136 |  |  | |
| NES | 1.020 (0.911, 1.142) | 0.734 |  |  | |
| EOS% | 0.954 (0.833, 1.094) | 0.502 |  |  | |
| BAS% | 0.710 (0.264, 1.913) | 0.498 |  |  | |
| LYM% | 0.970 (0.937, 1.003) | 0.076 |  |  | |
| MON% | 1.054 (0.952, 1.168) | 0.309 |  |  | |
| *Baseline body composition* | | | | |  |
| SMI (cm^2^/m^2^) | 0.938 (0.546, 1.611) | 0.815 |  |  | |
| VATI (cm^2^/m^2^) | 0.934 (0.541, 1.614) | 0.808 |  |  | |
| SATI (cm^2^/m^2^) | 0.949 (0.549, 1.639) | 0.851 |  |  | |
| SM-RA (HU) | 0.956 (0.548, 1.668) | 0.874 |  |  | |
| VAT-RA (HU) | 1.001 (0.581, 1.727) | 0.996 |  |  | |
| SAT-RA (HU) | 1.194 (0.693, 2.059) | 0.523 |  |  | |

| **Table S5. Characteristics of 48 lung cancer patients analyzed for peripheral blood lymphocyte** **abundance** | | | | | |
| --- | --- | --- | --- | --- | --- |
|  | Whole | | SMI stable | SMI loss | *p*-value |
| Age | 60 (53, 66) | | 60 (53, 66) | 61 (53, 64) | 0.806 |
| Sex (n (%)) |  | |  |  | 0.648 |
| female | 30 (62.5%) | | 12 (40.0%) | 6 (33.3%) |  |
| male | 18 (37.5%) | | 18 (60.0%) | 12 (66.7%) |  |
| BMI | 23.0 (20.9, 26.4) | | 23.6 (21.5, 26.9) | 21.7 (20.5, 24.7) | 0.125 |
| Pathology (n (%)) |  | |  |  | 0.557 |
| NSCLC | 39 (81.3%) | | 26 (86.7%) | 13 (72.2%) |  |
| SCLC | 9 (18.7%) | | 4 (13.3%) | 5 (27.8%) |  |
| Stage (n (%)) (1) |  | |  |  | 0.570 |
| III | 9 (19.1%) | | 5 (16.7%) | 4 (23.5%) |  |
| IV | 38 (80.9%) | | 25 (83.3%) | 13 (76.5%) |  |
| PD-L1 expression (n (%)) | | |  |  | 0.776 |
| negative | 7 (14.6%) | | 5 (16.7%) | 2 (11.1%) |  |
| positive | 15 (31.3%) | | 9 (30.0%) | 6 (33.3%) |  |
| unknown | 26 (54.1%) | | 16 (53.3%) | 10 (55.6%) |  |
| Regimen n (%) | |  |  |  | 0.103 |
| non-immunotherapy | | 26 | 19 | 7 |  |
| immunotherapy | | 22 | 11 | 11 |  |
| RECIST (n (%)) | |  |  |  | 0.238 |
| no PD | | 24 (50.0%) | 17 (56.7%) | 7 (38.9%) |  |
| PD | 24 (50.0%) | | 13 (43.3%) | 11 (61.1%) |  |
| NLR (n (%)) |  | |  |  | 0.161 |
| <3 | 23 (47.9%) | | 12 (40%) | 11 (57.9%) |  |
| ≥3 | 25 (52.1%) | | 18 (60%) | 7 (42.1%) |  |
| *Tumor markers* | | | | | |
| CEA | 6.1 (3.3, 19.9) | | 5.6 (3.1, 18.6) | 7.8 (3.8, 25.0) | 0.278 |
| NSE (3) | 18.3 (13.8, 26.2) | | 19.9 (13.8, 26.2) | 16.6 (13.3, 34.8) | 0.758 |
| CYFRA21-1 (3) | 5.3 (3.6, 10.8) | | 4.5 (3.3, 10.5) | 5.3 (4.3, 12.6) | 0.255 |
| *Peripheral blood lymphocyte classification* | | | | | |
| CD3+ (%) | 68.0 (60.9, 72.8) | | 67.9 (63.0, 74.5) | 68.0 (56.1, 71.8) | 0.431 |
| CD3+CD4+ (%) | 38.1 (33.8, 42.9) | | 38.4 (34.2, 41.4) | 36.5 (32.4, 43.4) | 0.898 |
| CD3+CD8+ (%) | 24.8 (19.4, 30.1) | | 26.7 (21.1, 35.8) | 21.6 (16.5, 27.9) | 0.056 |
| CD4+/CD8+ | 1.6 (1.1, 2.1) | | 1.5 (1.0, 1.9) | 1.9 (1.2, 2.6) | 0.145 |
| *Hematological parameters* | | | | | |
| WBC | 7.3 (5.8, 8.9) | | 7.4 (5.8, 9.4) | 6.9 (5.8, 8.0) | 0.418 |
| NEU% | 69.6 (61.7, 74.2) | | 70.0 (64.8, 75.3) | 68.0 (56.2, 74.1) | 0.343 |
| EOS% | 2.1 (0.8, 4.3) | | 1.8 (0.7, 3.6) | 2.7 (1.0, 4.8) | 0.418 |
| BAS% | 0.4 (0.3, 0.7) | | 0.5 (0.3, 0.7) | 0.4 (0.3, 0.6) | 0.465 |
| LYM% | 22.8 (16.4, 29.6) | | 22.3 (15.3, 28.1) | 24.6 (19.5, 31.8) | 0.221 |
| MON% | 4.7 (3.8, 5.7) | | 4.5 (3.8, 5.8) | 4.9 (4.3, 6.0) | 0.190 |
| RBC | 4.4 (4.1, 4.8) | | 4.5 (4.2, 4.9) | 4.3 (4.0, 4.7) | 0.282 |
| Hb | 134.5 (121.3, 144.8) | | 137.0 (120.5, 149.0) | 134.0 (118.8, 140.8) | 0.462 |
| Independent t-test or Mann-Whitney-U test was used for continuous variables. Fishers exact test when comparing categorical variables. Data are presented as median (IQR). CEA (ng/mL): Carcinoembryonic Antigen; NSE (ng/mL): Neuron-specific enolase; CYFRA21-1 (ng/mL): Cytokeratin 19; NLR: neutrophil/lymphocyte ratio; WBC (10^9/L): white blood cell; NEU%: neutrophil %; EOS%: eosinophils %; BAS%: basophil %; LYM%: lymphocyte %; MON%: monocyte %; RBC (10^9/L) : red blood cell; Hb (g/L): hemoglobin. | | | | | |

| **Table S6. Characteristics of 24 lung cancer patients analyzed for tumor immune cell infiltration** | | | | |
| --- | --- | --- | --- | --- |
|  | Whole | SMI stable | SMI loss | *p*-value |
| Age | 56 (52, 60.5) | 56 (55, 68) | 56 (51, 60) | 0.663 |
| Sex (n (%)) |  |  |  | 1.000 |
| female | 10 (41.7%) | 2 (40.0%) | 8 (42.1%) |  |
| male | 14 (58.3%) | 3 (60.0%) | 11 (57.9%) |  |
| BMI | 23.5 (22.4, 25.7) | 25.5 (25.1, 25.8) | 23.0 (22.0, 24.9) | **0.049** |
| Pathology (n (%)) |  |  |  | 0.557 |
| NSCLC | 16 (69.6%) | 2 (50.0%) | 14 (73.7%) |  |
| SCLC | 7 (30.4%) | 2 (50.0%) | 5 (26.3%) |  |
| Stage (n (%)) |  |  |  | 0.179 |
| II | 1 (4.2%) | 1 (20.0%) | 0 (0%) |  |
| III | 3 (12.5%) | 1 (20.0%) | 2 (10.5%) |  |
| IV | 20 (83.3%) | 3 (60.0%) | 17 (89.5%) |  |
| PD-L1 expression (n (%)) | |  |  | 0.185 |
| negative | 5 (20.8%) | 2 (40.0%) | 3 (15.8%) |  |
| positive | 7 (29.2%) | 0 (0%) | 7 (36.8%) |  |
| unknown | 12 (50.0%) | 3 (60.0%) | 9 (47.4%) |  |
| RECIST (n (%)) |  |  |  | 1.000 |
| no PD | 13 (54.2%) | 3 (60.0%) | 10 (52.6%) |  |
| PD | 11 (45.8%) | 2 (40.0%) | 9 (47.4%) |  |
| NLR (n (%)) |  |  |  | 0.615 |
| <3 | 15 (62.5%) | 4 (80.0%) | 11 (57.9%) |  |
| ≥3 | 9 (37.5%) | 1 (20.0%) | 8 (42.1%) |  |
| *Tumor markers* | | | | |
| CEA | 5.0 (2.0, 7.5) | 5.0 (2.9, 6.4) | 5.0 (2.0, 7.9) | 0.857 |
| NSE | 14.4 (11.3, 21.3) | 18.2 (10.6, 26.7) | 13.8 (11.9, 20.2) | 0.783 |
| CYFRA21-1 | 2.4 (1.9, 2.9) | 2.4 (2.1, 2.8) | 2.3 (1.9, 3.0) | 1.000 |
| *Multiplex immunofluorescence staining* | | | | |
| PD-L1% | 2.9 (1.9, 4.3) | 4.3 (2.9, 16.0) | 2.7 (1.9, 3.6) | 0.167 |
| CD3+% | 10.1 (5.3, 15.3) | 5.7 (3.1, 15.0) | 10.3 (6.7, 16.4) | 0.516 |
| CD3+CD4+% | 7.9 (4.8, 10.8) | 12.4 (4.4, 13.9) | 7.8 (5.3, 9.7) | 0.100 |
| CD3+CD8+% | 7.7 (3.8, 12.8) | 13 (4.4, 14.4) | 7.3 (3.8, 11.1) | **0.036** |
| CD3+CD4+FOXP3+% | 9.4 (3.7, 13.6) | 7.2 (4.0, 13.8) | 9.4 (3.5, 13.2) | 0.611 |
| *Hematological parameters* | | | | |
| WBC | 6.6 (5.2, 8.1) | 7.6 (5.6, 8.5) | 6.3 (5, 7.7) | 0.570 |
| NEU% | 64.7 (60.0, 72.2) | 67.4 (61.3, 68.9) | 64.1 (58.8, 77.1) | 0.683 |
| EOS% | 1.6 (0.9, 2.1) | 2.1 (0.9, 2.6) | 1.6 (0.8, 2.0) | 0.393 |
| BAS% | 0.4 (0.3, 0.5) | 0.5 (0.4, 0.7) | 0.4 (0.2, 0.5) | 0.210 |
| LYM% | 24.1 (17.5, 31.5) | 23.9 (23.2, 24.3) | 27.7 (13.1, 33.7) | 0.903 |
| MON% | 5.8 (4.8, 6.8) | 7.2 (5.2, 8.0) | 5.7 (4.8, 6.4) | 0.413 |
| RBC | 4.4 (3.7, 4.6) | 4.5 (4.4, 4.7) | 4 (3.6, 4.5) | **0.023** |
| Hb | 129 (110.5, 141.2) | 133 (130, 140) | 125 (106.5, 144.5) | 0.163 |
| Independent t-test or Mann-Whitney-U test was used for continuous variables. Fishers exact test when comparing categorical variables. Data are presented as median (IQR). CEA (ng/mL): Carcinoembryonic Antigen; NSE (ng/mL): Neuron-specific enolase; CYFRA21-1 (ng/mL): Cytokeratin 19; NLR: neutrophil/lymphocyte ratio; WBC (10^9/L): white blood cell; NEU%: neutrophil %; EOS%: eosinophils %; BAS%: basophil %; LYM%: lymphocyte %; MON%: monocyte %; RBC (10^9/L) : red blood cell; Hb (g/L): hemoglobin. | | | | |

| **Table S7. Nutrition Risk Screening Form for Adult Inpatients** | | | | | | |
| --- | --- | --- | --- | --- | --- | --- |
| Name: | | Sex: | Department: | Bed No.: | ID: | |
| Scoring Items | | | | | | Score |
| Nutritional Status | 0 points – Normal nutritional status | | | | |  |
|  | 1 point – Weight loss >5% in 3 months; or 50–75% food intake in past week | | | | |  |
|  | 2 points – Weight loss >5% in 2 months; or 25–50% food intake in past week | | | | |  |
|  | 3 points – Weight loss >5% in 1 month or >15% in 3 months; or BMI <18.5 with poor condition; or 0–25% food intake in past week | | | | |  |
| Disease Severity | 0 points – Normal nutritional requirement | | | | |  |
|  | 1 point – Mild increase: hip fracture, chronic diseases with acute exacerbation (e.g., cirrhosis, COPD, dialysis, diabetes, malignancy) | | | | |  |
|  | 2 points – Moderate increase: major abdominal surgery, stroke, severe pneumonia, hematologic malignancies | | | | |  |
|  | 3 points – Severe increase: TBI, bone marrow transplant, ICU (APACHE >10) | | | | |  |
| Age | 0 points – 18–69 years | | | | |  |
|  | 1 point – ≥70 years | | | | |  |
| Total Nutrition Risk Score | | | | | |  |
| Remarks:  1. Total Nutrition Risk Score = sum of all three items above.  2. Score ≥3: Patient is at nutritional risk – nutrition therapy is required. Please consult the nutrition department.  3. Score <3: Rescreen in one week. If major surgery is scheduled, consider preventive nutritional therapy to avoid combined risks. | | | | | | |


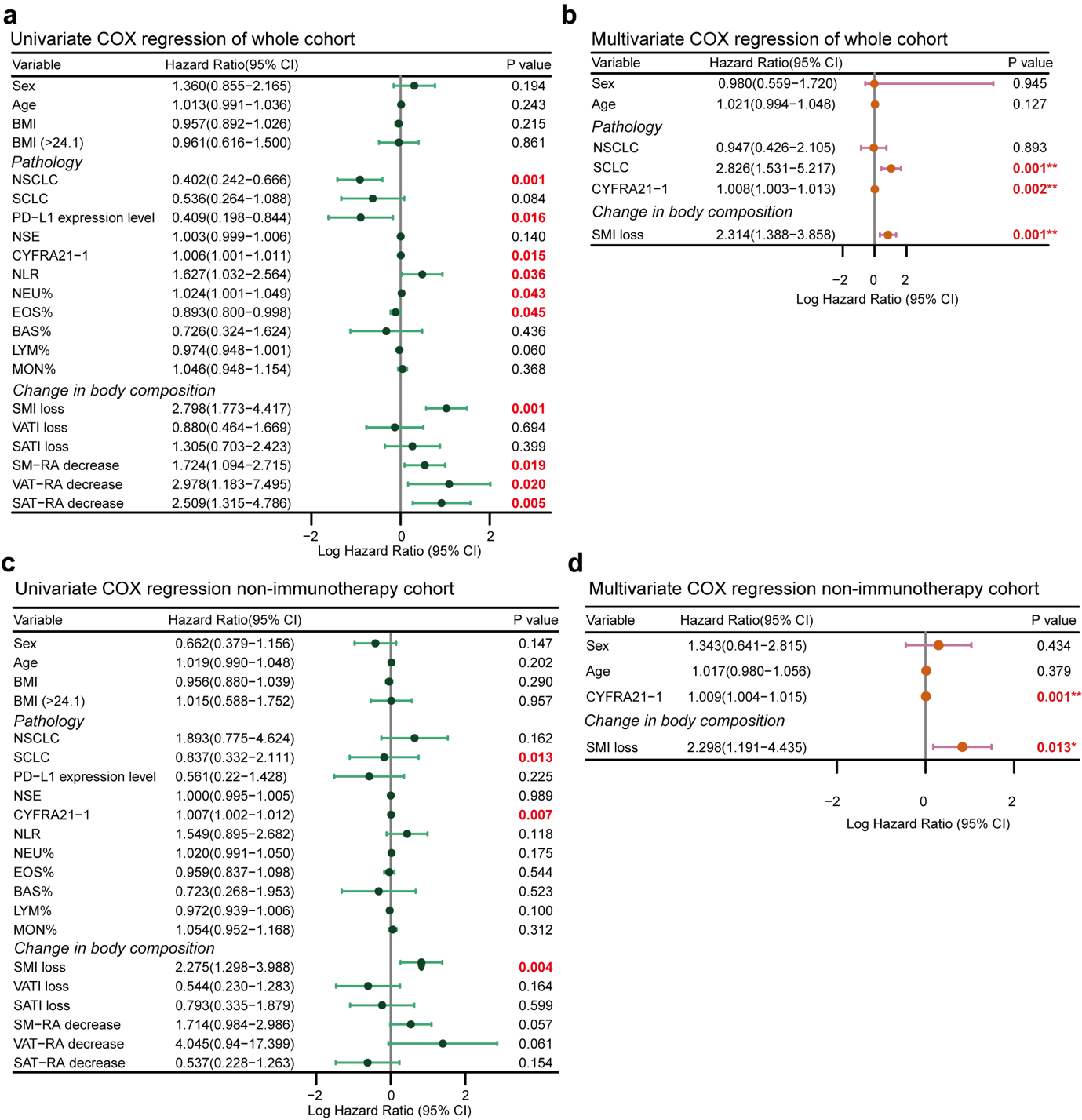


**Figure S1. Cox proportional hazard models for the association of various parameters with OS in the whole and non-immunotherapy cohort.** (a and c) Univariate Cox regression analysis; (b and d) Multivariate Cox regression analysis.


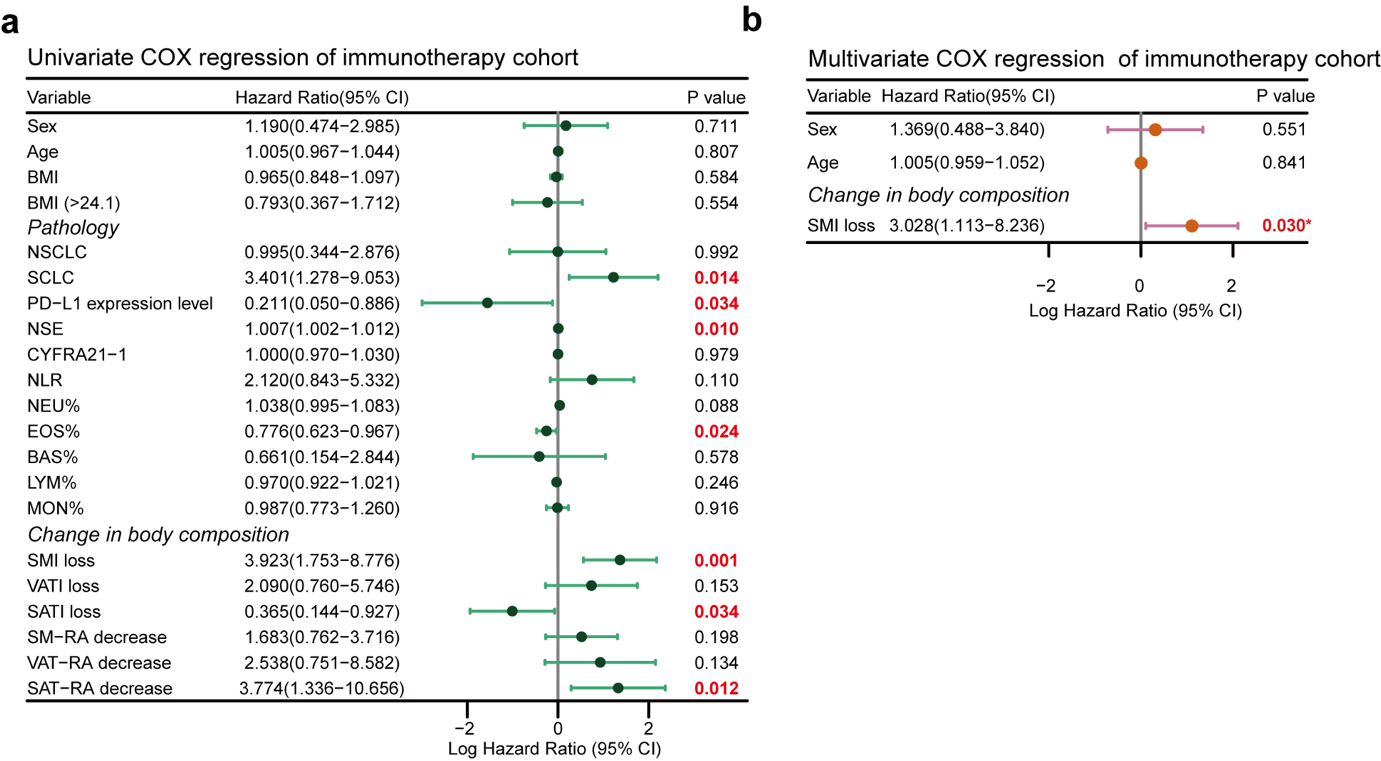


**Figure S2. Cox proportional hazard models for the association of various parameters with OS in the immunotherapy cohort.** (a) Univariate Cox regression analysis; (b) Multivariate Cox regression analysis.


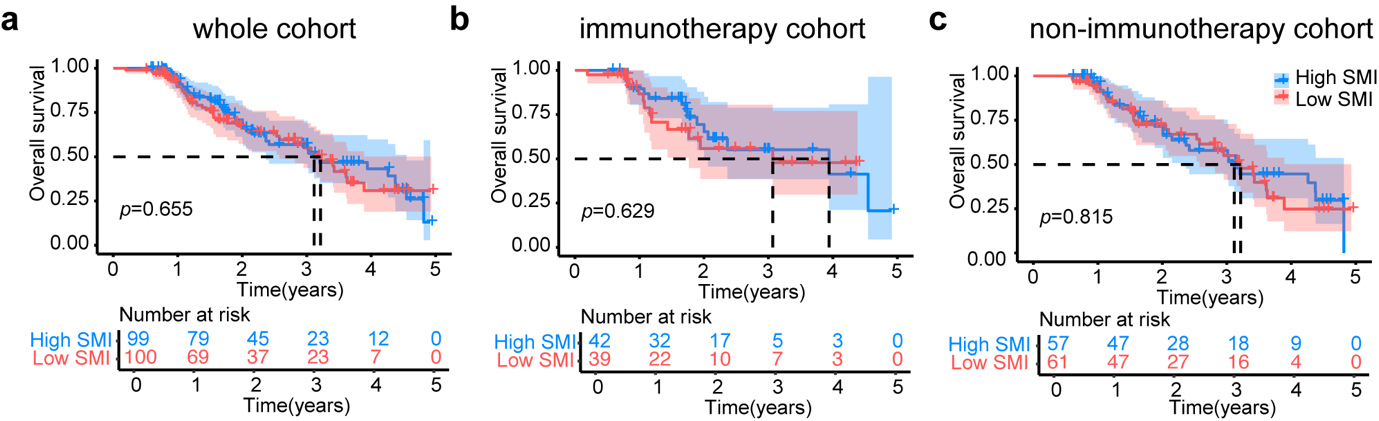


**Figure S3. Prognostic differences among patients with different high SMI and low SMI.** The Kaplan-Meier curves represent the overall survival time in (a) whole cohort, (b) immunotherapy cohort, and (c) non-immunotherapy cohort (one patient’s survival data was unavailable).


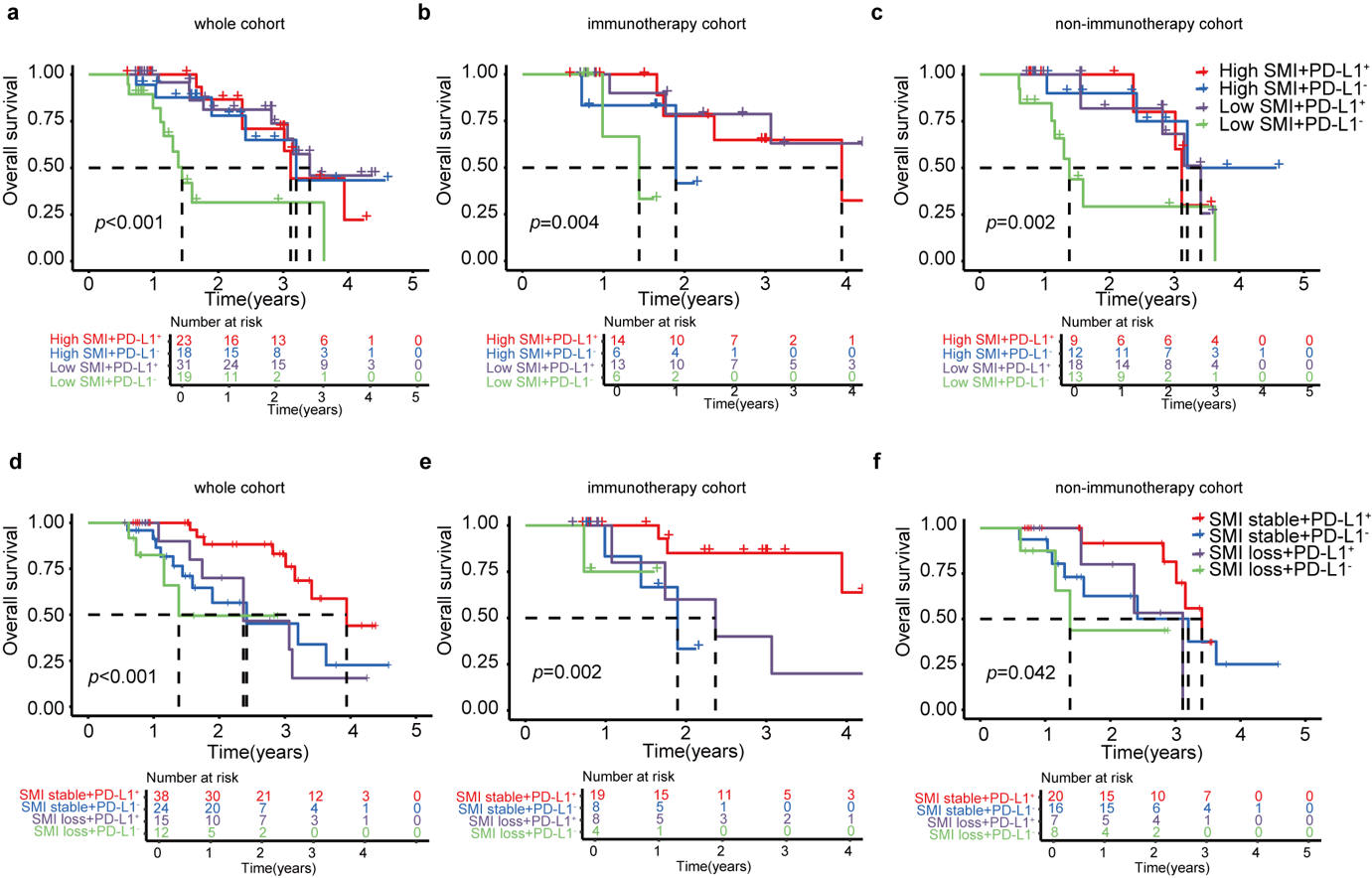


**Figure S4. Survival analysis according to baseline SMI or SMI change during therapy combined with PD-L1 expression status.** Kaplan-Meier curves of baseline SMI combined with PD-L1 expression status in the whole cohort (a), immunotherapy cohort (b), and non-immunotherapy cohort (c). Kaplan-Meier curves of SMI change combined with PD-L1 expression status in the whole cohort (d), immunotherapy cohort (e), and non-immunotherapy cohort (f).


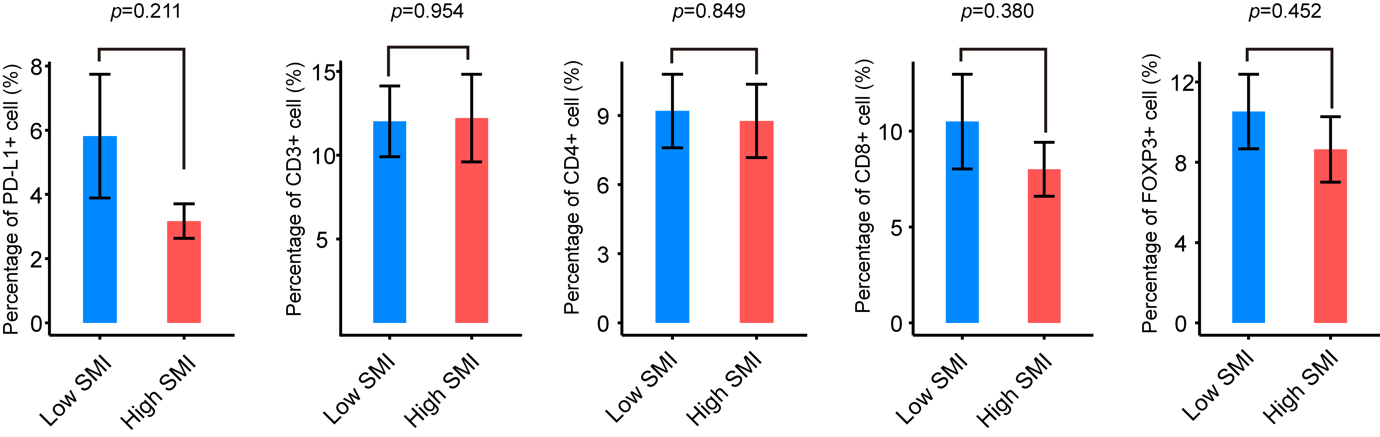


**Figure S5. The percentages of different TILs in patients with low versus high pre-treatment skeletal muscle mass.** The percentages of cells in the tumor microenvironment expressing the indicated markers are shown for patients with low versus high SMI at baseline.
